# Genomic dissection of the antimicrobial resistance epidemiology of *Salmonella* Typhimurium

**DOI:** 10.3389/fmicb.2025.1683995

**Published:** 2025-11-20

**Authors:** Sandeep Kaur, Sally R. Partridge, Michael Payne, Vitali Sintchenko, Ruiting Lan

**Affiliations:** 1 School of Biotechnology and Biomolecular Sciences, The University of New South Wales, Sydney, NSW, Australia; 2 Centre for Infectious Diseases and Microbiology, The Westmead Institute for Medical Research and Westmead Hospital, Westmead, NSW, Australia; 3 Faulty of Medicine and Health, The University of Sydney, Sydney, NSW, Australia; 4 Sydney Infectious Diseases Institute, The University of Sydney, Sydney, NSW, Australia

**Keywords:** antimicrobial resistance, multilevel genome typing, genomic surveillance, *Salmonella* Typhimurium, ciprofloxacin, cefotaxime

## Abstract

**Introduction:**

*Salmonella* Typhimurium (STm) is a globally distributed foodborne pathogen showing increasing antimicrobial resistance (AMR), particularly to fluoroquinolones and third-generation cephalosporins. Multiple countries have implemented ongoing genomic surveillance programs for *Salmonella*, but comprehensive global analyses integrating genomic typing and AMR in STm remain scarce.

**Methods:**

Publicly available genomes of ~65,000 STm isolates were characterized using Multilevel Genome Typing (MGT). Resistance was predicted to 14 clinically-relevant antibiotics. Resistance patterns were analyzed by MGT sequence type (ST), geographic location, year of collection, and source. MGT ST where ≥80% isolates were predicted to be resistant to an antibiotic were defined as a resistant ST for that antibiotic.

**Results:**

About half of all STm isolates were predicted to be resistant to ≥1 antibiotic. Resistance frequencies varied substantially by country, collection year and MGT ST, and 407 resistant MGT STs were identified. Among the most recent isolates (2021–2022), eight MGT STs were classified as cefotaxime resistant and three as ciprofloxacin intermediate. Cefotaxime resistant MGT STs predominantly included isolates from cattle/poultry in the USA. Ciprofloxacin intermediate MGT STs were mainly linked to swine from the UK.

**Discussion:**

This large-scale genomic analysis highlights substantial diversity in AMR patterns among STm genomic types globally. The identification of recently emerged cefotaxime resistant and ciprofloxacin intermediate STs underscores the continued threat of resistance to antibiotics critical for treatment of severe salmonellosis. Integration of MGT strain typing with AMR prediction provides scalable, sharable, standardised and precise tracking of resistant isolates/STs, offering a powerful framework for global AMR surveillance.

## Introduction

*Salmonella enterica* serovar Typhimurium (STm) is one of the two most frequently isolated non-typhoidal *Salmonellae* (NTS) serovars identified globally (Williamson et al., 2018; Department of Health and Aged Care, Australian Government, 2023). NTS, especially STm, have a broad host range and infect humans through direct contact with other hosts or consumption of contaminated food (Ehuwa et al., 2021; Branchu et al., 2018). NTS infect the gastrointestinal tract, and disease severity is generally mild to moderate. However, NTS can also result in bacteraemia, and cause endocarditis, abscesses, or meningitis (Fierer, 2022). Antibiotics are not recommended for mild to moderate cases of salmonellosis in healthy individuals but are required in severe cases, with fluoroquinolones (e.g., ciprofloxacin) recommended as first-line treatment in adult travellers by the US CDC (Plumb et al., 2024). Ceftriaxone (third-generation cephalosporin) or azithromycin (macrolide) are recommended for adults with invasive disease and for children. STm is dominated by four multi-locus sequence types (STs), ST19 (the most prevalent), ST34, ST313 and ST36 (Achtman et al., 2012). The monophasic variant of STm generally belongs to ST34 (Achtman et al., 2012). Two multidrug resistant lineages (L1 and L2) associated with invasive disease in Africa (Van Puyvelde et al., 2019) belong to ST313. ST36 forms a distinct phylogenetic group from other STs (Bawn et al., 2020; Gymoese et al., 2017).

In previous work, we developed multilevel genome typing (MGT) for characterising STm isolates at multiple levels of resolution (Payne et al., 2020). Briefly, the MGT scheme consists of nine MLST-like schemes that increase in resolution from MGT1 to MGT9. A set of nine MGT STs, one for each level, are assigned to each isolate and, using these nine STs, evolutionary relationships between isolates can be studied at multiple levels of resolution (such as global, local, long-term or short-term). MGT1 STs correspond to those from the established 7-gene MLST for *Salmonella* (Achtman et al., 2012) e.g., ST19 or ST34. MGT8 comprises the species core genome MLST (cgMLST) (Payne et al., 2020). MGT2 to 7 are composed of increasing numbers of mutually-exclusive sets of core genes from MGT8. MGT9 is the serovar core genome MLST (Payne et al., 2020). Since MGT levels 1 to 7 are based on mutually exclusive sets of loci, a higher-level MGT ST may comprise isolates from multiple lower-level MGT STs (Payne et al., 2020). Many well studied clades and types can be described using intermediate resolution levels. For example, MGT3 ST11 corresponds to phage type DT104, a global pandemic clone, while two large groups composed mainly of avian isolates from the USA [Foodborne Disease Burden Epidemiology Reference Group (FERG), Monitoring and Surveillance Nutrition and Food Safety (MNF), Nutrition and Food Safety (NFS), 2023] correspond to MGT3 ST8 and MGT3 ST171 (Payne et al., 2020). Monophasic STm belong to MGT3 ST26, MGT3 ST18 or MGT3 ST225, all within MGT1 ST34. Lineages L1 and L2 in MGT1 ST313 correspond to MGT3 ST725 and MGT3 ST3, respectively (Payne et al., 2020).

The emergence of antimicrobial resistance (AMR) in STm has been well documented (Branchu et al., 2018; Baker et al., 2018). For example, ST34 isolates often have the ASSuT resistance pattern (i.e., to **a**mpicillin, **s**treptomycin, **su**lphathiazole, and **t**etracycline). The corresponding genes, *bla*_TEM-1_, *strAB* [also called *aph(3″)-Ib* and *aph(6)-Id*], *sul2*, and *tet*(B), are present on a chromosomal resistance island (Lucarelli et al., 2012). ST34 isolates may also be resistant to additional drugs, such as quinolones, cefotaxime, colistin, gentamicin, and chloramphenicol (Sun et al., 2020). The ACSSuT resistance pattern (the additional ‘C’ is **c**hloramphenicol) has been found in a large proportion of phage type DT104, or MGT3 ST11, isolates. The *bla*_CARB-2_, *floR*, *aadA2*, *sul1* and *tet*(G) genes, respectively, found in chromosomal *Salmonella* genomic island 1 (SG1) (Monte et al., 2020; Mulvey et al., 2006; Briggs and Fratamico, 1999), are often responsible for this phenotype. However, variations in the gene content of SG1 have been observed in both STm and other serovars (Mulvey et al., 2006). DT104 isolates may also be resistant to additional drugs such as gentamicin, kanamycin, and trimethoprim-sulphamethoxazole (Besser et al., 2000). DT104 was considered a pandemic strain from 1990 to 2010, whereas ST34 (or DT193/DT120) is considered the current pandemic strain (Branchu et al., 2018). The prevalent lineage associated with bloodstream infections, ST313 L2, corresponding to MGT3 ST3, has well known resistance to multiple drugs such as ampicillin, trimethoprim-sulfamethoxazole and chloramphenicol, with reports also emerging of resistance to azithromycin and cephalosporins (Van Puyvelde et al., 2019).

In recent years, whole genome sequencing (WGS) has been adopted for the long term surveillance of *Salmonella* in the UK (Ashton et al., 2016) and the USA (Centers for Disease Control and Prevention, 2022), with complete WGS surveillance datasets available publicly. Given this, we conducted a comprehensive survey of predicted AMR in STm, applying MGT. We demonstrate the utility of the standardised and multi-resolution MGT approach to precisely identify groups of resistant strains, especially those with resistance to antibiotics important for treatment of human STm infections, and determined global temporal trends of AMR MGT STs.

## Materials and methods

### Genome assembly and assignment of MGT STs

We previously developed and maintain a database (MGTdb)^1^ for typing STm isolates using MGT (Payne et al., 2020; Kaur et al., 2022) that includes the following pipeline: every day, the sequences of any new isolates available in NCBI SRA (Cochrane et al., 2016), particularly Illumina paired-end sequences, are imported into MGTdb. These, along with any sequence data uploaded directly to MGTdb by users, are assembled with a pipeline that uses SKESA (Souvorov et al., 2018). Alleles are extracted from the assemblies and used to type the isolates at all MGT levels (Payne et al., 2020). The isolate ‘Run’ identifiers imported from NCBI SRA are made publicly available, and the information about isolates uploaded by users can be set by the user to be either ‘public’ or ‘private’. As automated AMR prediction is not yet implemented in MGTdb and due to the scale of analysis in this study, we analysed all isolates with publicly available sequence data in MGTdb until August 31, 2023 that were typed at one or more MGT levels. The sample collection dates in NCBI SRA associated with the isolates are imported into MGTdb, and the collection year was used in the analysis of trends in this work.

### Annotation of AMR mechanisms

We used abritAMR (Sherry et al., 2023) (version 1.0.14) with default settings to assign AMR profiles for each isolate from assembled sequence data, identifying AMR determinants in 16 resistance categories broadly corresponding to different antibiotics, although some categories are based on gene type detected. We then used the ‘report’ function in abritAMR to generate predicted phenotypic interpretations for these categories. abritAMR reports “cefotaxime (AmpC)” and “cefotaxime (ESBL)” separately, as the phenotypic profiles are different, but these were merged into a single “cefotaxime” category here, as both AmpC and ESBL mechanisms confer resistance to this antibiotic. Additionally, abritAMR identifies colistin resistance mechanisms and 16S rRNA methylase genes (aminoglycoside resistance, e.g., *armA*, *rmt*) but does not provide phenotypic interpretations. For consistency in the presentation of results (resistance mechanisms grouped into antibiotic classes), we classified isolates carrying any colistin resistance mechanism as “resistant” to colistin, unless otherwise specified in the results (e.g., the *mcr-9* gene for colistin resistance was marked as predicting a susceptible phenotype). We excluded 16S rRNA methylase genes from further analysis due to lack of validation of the phenotypic prediction of resistance based on this set of genes.

### Validation of AMR prediction

To validate abritAMR’s phenotypic interpretations based on genotypic predictions of resistance, we analyzed isolates from NCBI’s Pathogen Detection resource (NCBI, 2016) up to 14 December, 2022 that had both phenotypic antimicrobial susceptibility testing (AST) data and Illumina paired-end sequence data. For consistency in comparison, isolates were classified into two categories: susceptible or resistant (including intermediate), based on either AST results or genomics-based predictions.

For each antibiotic, we defined the following metrics:

- True positives (TP): isolates predicted as resistant or intermediate that are phenotypically resistant or intermediate.
- True negatives (TN): isolates predicted as susceptible that are phenotypically susceptible.
- False positives (FP): isolates predicted as resistant or intermediate that are phenotypically susceptible.
- False negatives (FN): isolates predicted as susceptible that are phenotypically resistant or intermediate.

We then calculated accuracy using the formula:


$$\text{Accuracy}=\frac{\mathrm{TP}+\mathrm{TN}}{\mathrm{TP}+\mathrm{TN}+\mathrm{FP}+\mathrm{FN}}$$

### Resistance score metrics

For each isolate, we represented susceptibility to an antibiotic as 0 and resistance (including intermediate resistance in the case of ciprofloxacin) as 1. A resistance score ($R_{s}$) was then calculated as the sum of these Boolean values across the 14 antibiotics.

For any subset of isolates, we computed:

- $\bar{R_{s}}$: The mean resistance score of all isolates in the subset.
- $\bar{R_{s,R}}$: The mean resistance score of isolates that were resistant to at least one antibiotic.

To assess variability, bootstrapping was performed 9,999 times using Python (version 3.9.6) and the scipy.stats package (version 1.10.1) (SciPy Documentation, 2023) for the datasets specified in the results. Statistical comparisons of $\bar{R_{s}}$ and $\bar{R_{s,R}}$ between different data subsets were conducted using the Mann–Whitney *U* test (McKnight & Najab, 2010), implemented in the scipy.stats package (version 1.10.1) (SciPy Documentation, 2023) in python3 (version 3.9.6) (with continuity set to False, and significance tested at a Bonferonni corrected *p*-value (VanderWeele and Mathur, 2019) as indicated in the results).

### Comparison of resistance to individual antibiotics

To compare resistance to individual antibiotics between data subsets (where at least one subset contained ≥10 isolates), we used the Binomial test (Abdi, 2007). Susceptibility was assigned a value of 0, while resistance (including intermediate resistance to ciprofloxacin) was assigned 1. We then compared the probability of resistance/intermediate resistance between subsets independently. Significance was tested at the Bonferroni corrected *p*-value as indicated in the results. The Binomial test used was implemented in the scipy.stats package (version 1.10.1) (SciPy Documentation, 2023) in python3 (version 3.9.6).

### Definition of MGT STs as resistant (or intermediate for ciprofloxacin)

We defined resistant MGT STs (plus intermediate STs for ciprofloxacin) as those in which ≥80% of isolates were resistant (or intermediate) to a given antibiotic. To determine these, we traversed MGT levels 1 to 9, calculating the percentage isolates predicted to be resistant (or intermediate in the case of ciprofloxacin) at each level. At each step (i.e., MGT level), MGT STs meeting the ≥80% threshold were classified as resistant or ciprofloxacin-intermediate and not analysed further. The process was then repeated at the next MGT level, using only the remaining isolates (Supplementary Figure S7). This iterative approach ensured that each isolate was assigned to only one resistant/intermediate MGT ST, preventing duplication across different levels (Supplementary Figure S7). This analysis was performed in python3 (version 3.9.6).

Prior to settling on a threshold of 80%, we assessed the performance of different thresholds (50%, 60%, 70%, 80%, and 90%) to define resistant (or ciprofloxacin-intermediate) MGT STs, defining the following metrics for each antibiotic:

- True positives (TP): resistant or ciprofloxacin-intermediate isolates included within resistant or ciprofloxacin-intermediate MGT STs.
- True negatives (TN): susceptible isolates excluded from resistant or ciprofloxacin-intermediate MGT STs.
- False positives (FP): susceptible isolates included within resistant or ciprofloxacin-intermediate MGT STs.
- False negatives (FN): resistant isolates excluded from resistant or ciprofloxacin-intermediate MGT STs.

We then calculated precision, recall and F1 scores as:


$$\text{Precision}=\frac{\mathrm{TP}}{\mathrm{TP}+\mathrm{FP}}$$

$$\text{Recall}=\frac{\mathrm{TP}}{\mathrm{TP}+\mathrm{FN}}$$

$$F1\;\text{score}=\frac{2*\text{Precision}*\text{Recall}}{\text{Precision}+\text{Recall}}$$

### Temporal clusters

For a given antibiotic, we standardized each MGT ST analyzed using the counts per year, as:


$$(x_{i}-m)/s$$

Where *x_i_* is the number of isolates in an MGT ST in one year, *m* is the mean count across all years, and *s* is the standard deviation. We used silhouette index (Rousseeuw, 1987) to initially identify the best (i.e., highest silhouette index) number of clusters for a dataset, then manually inspected the separation between the clusters to select the final number of clusters. Analyses were performed in R (version 4.3.1), using the bios2mds package (Pelé et al., 2012) (version 1.2.3) to calculate the silhouette index, and the Mfuzz package (Kumar & Futschik, 2007) (version 2.60.0) to perform temporal clustering. We used a custom function in R to plot the resulting clusters. The clusters were manually rearranged, in order of decreasing initial weighted mean.

### Resistance or ciprofloxacin-intermediate mechanism clustering

Within an MGT ST, for a given antibiotic, we calculated the percentage of isolates that were susceptible, and the percentage of isolates with each resistance mechanism, or combination of mechanisms. This set, comprising all resistance mechanisms for a given antibiotic, and the empty set (indicating susceptibility to the antibiotic) was termed *M*. Then, we calculated the distance between any two MGT STs *i* and *j* as:


$$\sum_{m=1}^{M}\text{minimum}(p_{i},p_{j})$$

Where, *p1* and *p2* are percentage of isolates in the two MGT STs, respectively, with *m* ∈ *M*.

Once this similarity matrix was calculated, we utilized the clustermap function in the seaborn library (version 0.12.2) in python3 (version 3.9.6), to perform unweighted pair group method with arithmetic mean.

### MGT hierarchy

We generated a MGT hierarchy for each MGT ST defined as resistant or ciprofloxacin-intermediate in 2021–2022. For each MGT ST, the lower level MGT ST was displayed if at least 50% of the isolates were assigned the same ST at the lower MGT level, otherwise the MGT ST was indicated with an ‘X’. To infer the presence of plasmid replicons, PlasmidFinder (Carattoli et al., 2014) (downloaded on 2023, June 4) was run on each sequence using default settings (coverage 60%, identity 95%). For each MGT ST defined as resistant or ciprofloxacin-intermediate, a plasmid replicon type was annotated if the replicon was present in at least 80% of the isolates in the ST. We also displayed up to the top five sources of the isolates within an ST, where available after simplifying the source names. For example, “Human, stool” was simplified to “human”, “ground turkey” was simplified to “turkey”. The MGT hierarchy was generated in python3 (version 3.9.6) in dot format, which was then converted to a scalable vector graphic format using graphviz’s (version 8.0.5) hierarchical layout engine (Gansner, 2009).

## Results

### Dataset and resistance prediction

We previously developed and maintain a database (MGTdb) for typing STm isolates using MGT (Payne et al., 2020; Kaur et al., 2022). As of August 2023, our dataset consisted of 65,895 publicly available genomes in MGTdb that were assigned an ST at one or more MGT levels. The isolates span 94 countries and were sampled between 1900 and 2022. Since around 2013, the number of isolates with WGS data available sharply increased (Supplementary Figure S1). In this dataset, over half of the isolates have metadata available (Table 1), 63% with country, 59% with year, and 58% with both country and year specified, with 73% of the latter from the USA or the UK.

**Table 1:** Number of isolates from top 10 countries in descending order.

Country	With country		With country and year		2015–2018		2019–2022	
	Count	%	Count	%	Count	%	Count	%
USA	15,643	37.9	13,519	35.1	4,791	32.5	5,216	42.2
United Kingdom	15,117	36.6	14,733	38.3	7,127	48.4	5,066	41
Canada	1737	4.2	1,692	4.4	840	5.7	130	1.1
Australia	1,557	3.8	1,557	4	494	3.4	121	1
Denmark	1,148	2.8	1,141	3	31	0.2	4	0
South Africa	1,049	2.5	1,049	2.7	32	0.2	992	8
Germany	959	2.3	959	2.5	544	3.7	96	0.8
Taiwan	483	1.2	483	1.3	115	0.8	1	0
Italy	332	0.8	332	0.9	88	0.6	3	0
Japan	319	0.8	319	0.8	136	0.9	0	0
Total displayed	38,344	92.9	35,784	93	14,198	96.4	11,629	94.1
Total overall	41,285		38,482		14,738		12,361	
%	62.7		58.4		22.4		18.8	

The UK adopted genomic surveillance for *Salmonella* from 2015 (Ashton et al., 2016), and the USA from 2019 (Centers for Disease Control and Prevention, 2022). To provide a comparison between USA and UK that is potentially less biased by sampling effects, we considered a subset of data from 2019 to 2022, and three locations: the UK, the USA and other countries (designated ‘Other’). Additionally, as surveillance in the UK began earlier, we also compared UK data from 2015 to 2018 to the more recent UK data (2019–2022). We also considered data from 2015 to 2018 for the USA and the Other set. While surveillance in the USA did not begin until 2019, trends in the USA from 2015–2018 are likely to be reliable due to the large number of samples. However, in the Other set, trends cannot be considered reliable due to varied sampling (i.e., heterogeneous temporal and geographical coverage of isolates), and hence, we are cautious about discussing trends from these countries.

AMR mechanisms were identified from genome assemblies using abritAMR (Sherry et al., 2023), which detects relevant genes or mutations and, for *Salmonella*, predicts phenotypic resistance or susceptibility to corresponding antibiotics. Both, the phenotype predictions and the underlying resistance mechanisms for the 65,859 isolates are available in the MGT database (see text footnote 1). Partial genes with up to 10% of the sequence missing were also included in resistance predictions. For *Salmonella* spp., abritAMR is validated for prediction of resistance or susceptibility to 12 clinically relevant antibiotics (ampicillin, cefotaxime, meropenem, gentamicin, kanamycin, streptomycin, sulfathiazole, trimethoprim, trimethoprim-sulfathiazole, tetracycline, chloramphenicol, azithromycin), while predictions for a thirteenth, ciprofloxacin, are more complex. Most single mutations or acquired genes confer only low levels of resistance to ciprofloxacin. From comparing genotypes and phenotypes during validation of abritAMR, the best correlation was obtained by coding isolates with the absence of any ciprofloxacin resistance mechanism as susceptible, those with as single mechanism as intermediate and those with two or more as resistant (Sherry et al., 2023). We use this scheme here, but acknowledge that this may represent an oversimplification of the complexities involved in the genotype to phenotype prediction of ciprofloxacin resistance (Conley et al., 2018). Such simplification can occasionally result in incorrect phenotype prediction, an example being the presence of the *oqxA* and *oqxB* genes (encoding the OqxAB efflux pump) resulting in a resistant rather than intermediate classification by abritAMR (Li et al., 2019) (see Supplementary Results for details). Classification as ciprofloxacin-intermediate by abriTAMR implies that an isolate is likely to have a borderline MIC and different MIC cutoffs are used in different clinical standards (see Supplementary Results for details), so we did not modify outputs from abritAMR (for which phenotypic predictions were validated by the developers against the CLSI 2020 recommended values) (Sherry et al., 2023), and use them as provided by the tool.

Colistin resistance determinants (219 isolates, Supplementary Table S1) and 16S rRNA methylase genes, conferring high-level aminoglycoside resistance (44 isolates, Supplementary Table S2), were also identified, although phenotypic interpretations are not provided by abritAMR. Isolates carrying known colistin resistance mutations or genes [excluding *mcr-9* (Tyson et al., 2020)] were classified as resistant here and included in the analysis presented below. While 16S rRNA methylases are typically associated with high-level resistance to multiple aminoglycosides (Krause et al., 2016), limited phenotypic data precluded validation, and these mechanisms were excluded from the analysis below. Lastly, to independently assess abritAMR’s prediction accuracy, we compared abritAMR results with phenotypic AST data from 952 STm isolates available through NCBI Pathogen Detection (NCBI, 2016). Genotypic predictions showed 98.4% overall concordance with AST results across 12 antibiotics (Supplementary Table S3). Additional details of resistance mechanism interpretation and validation are provided in the Supplementary Results.

### Predicted resistance to any antibiotic, including intermediate resistance to ciprofloxacin

Based on these predictions, we calculated a resistance score ($R_{s}$) as the number of the 14 antibiotics an isolate is predicted to be resistant to (intermediate/resistant for ciprofloxacin). Then, for all isolates in a given set, we calculated the mean resistance score $\bar{(R_{s}}$), the number of isolates resistant (intermediate/resistant for ciprofloxacin) to at least one antibiotic (R), the resistance score of isolates in this set ($R_{s,R}$), and the mean of resistance score of isolates in this set ($\bar{R_{s,R}}$). Approximately half of the isolates (47%, $\bar{R_{s}}$= 2, and $\bar{R_{s,R}}$= 4.2) were resistant to at least one antibiotic, or intermediate/resistant to ciprofloxacin, but this varied by both location and time (Table 2). From 2015 to 2022 in the three locations, isolates from the UK had the highest resistance, with 60% resistant to at least one antibiotic ($\bar{R_{s}}$= 2.4, $\bar{R_{s,R}}$= 4), 45% of USA isolates were resistant to at least one antibiotic ($\bar{R_{s}}$= 1.5, $\bar{R_{s,R}}$= 3.4), and 42% of isolates from other locations were resistant to at least one antibiotic ($\bar{R_{s}}$= 1.8, $\bar{R_{s,R}}$= 4.2). Further comparisons of the proportions of resistant or ciprofloxacin intermediate/resistant isolates from the UK and the USA between 2015–2018 and 2019–2022, as well as between the UK and the USA in 2019–2022 are detailed in the Supplementary Results.

**Table 2:** Resistance scores in the complete dataset and in subsets of isolates.

Resistance	Overall		UK				USA				Other			
Score			2015–2018		2019–2022		2015–2018		2019–2022		2015–2018		2019–2022	
	Count	%	Count	%	Count	%	Count	%	Count	%	Count	%	Count	%
0	35,015	53.1	2,600	36.5	2,296	45.3	2,777	58.0	2,734	52.4	1,491	50.4	1,585	67.0
1	3,271	5	638	9	337	6.7	134	2.8	150	2.9	189	6.4	138	5.8
2	3,853	5.8	188	2.6	160	3.2	804	16.8	979	18.8	142	4.8	46	1.9
3	2,412	3.7	482	6.8	331	6.5	104	2.2	95	1.8	141	4.8	181	7.7
4	10,575	16	2065	29	1,182	23.3	560	11.7	868	16.6	451	15.2	148	6.3
5	3,133	4.8	382	5.4	287	5.7	171	3.6	153	2.9	171	5.8	82	3.5
6	3,978	6	294	4.1	131	2.6	140	2.9	116	2.2	118	4.0	50	2.1
7	1,626	2.5	236	3.3	113	2.2	36	0.8	48	0.9	82	2.8	36	1.5
8	967	1.5	153	2.1	75	1.5	29	0.6	22	0.4	92	3.1	53	2.2
9	667	1	64	0.9	136	2.7	18	0.4	30	0.6	39	1.3	37	1.6
10	299	0.5	13	0.2	15	0.3	11	0.2	17	0.3	30	1.0	3	0.1
11	68	0.1	8	0.1	2	0	4	0.1	4	0.1	10	0.3	3	0.1
12	31	0	4	0.1	1	0	3	0.1	0	0	3	0.1	2	0.1
13	0	0	0	0	0	0	0	0	0	0	0	0	0	0
14	0	0	0	0	0	0	0	0	0	0	0	0	0	0

### Predicted resistance to individual antibiotics, and intermediate to ciprofloxacin

**Figure 1:**
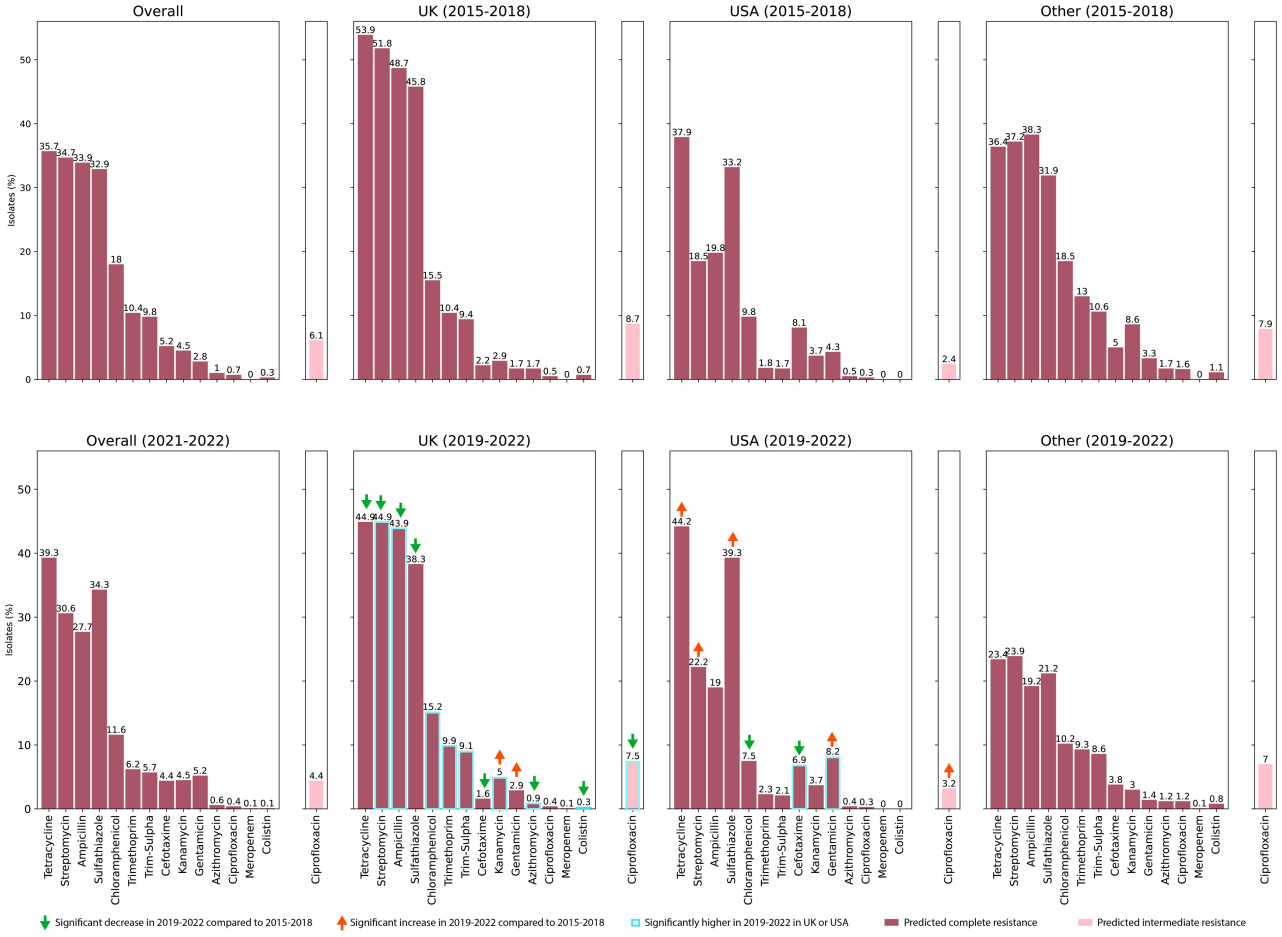
Predicted resistance to 14 antibiotics in the complete dataset and various subsets. Antibiotics are arranged from left to right in descending order of the number of resistant isolates, as observed in the overall dataset, except for colistin, which is shown last due to the lack of validated resistance prediction for this antibiotic by abritAMR. For isolates from the UK and the USA, significantly higher resistance in 2019–2022 compared to 2015–2018 is indicated by red arrows while significantly lower resistance is indicated by green arrows. A blue outline around a bar highlights significantly higher resistance during 2019–2022, comparing the UK and the USA. The differences in proportions of resistant and ciprofloxacin-intermediate isolates between subsets were assessed using the Binomial test, with significance assessed at a Bonferroni-corrected threshold of *p* < 0.0008.

Predicted resistance to any individual antibiotic in the complete dataset varied from 0.02% isolates for meropenem to 36% for tetracycline (Figure 1). The UK 2015–2018 dataset had some of the highest proportions of resistant isolates, especially to tetracycline (54%), streptomycin (52%), ampicillin (49%) and sulfathiazole (46%). Differences between the two time periods, 2015–2018 and 2019–2022, were compared in the UK and USA and statistically assessed using the Binomial test, with significance assessed at a Bonferroni-corrected threshold of *p* < 0.0008. In the UK there was a significant increase in the proportion of resistant isolates for only two antibiotics, kanamycin (from 2.9% to 5%) and gentamicin (from 1.7% to 2.9%) in 2019–2022 compared to 2015–2018, and a significant reduction in the proportion of isolates resistant to seven antibiotics, tetracycline (54% to 45%), streptomycin (52% to 45%), ampicillin (49% to 44%), sulfathiazole (46% to 38%), cefotaxime (2.2% to 1.6%), azithromycin (1.7% to to 0.9%) and colistin (0.7% to 0.3%) (Figure 1). In the USA, resistance to tetracycline (38% to 44%), streptomycin (19% to 22%), sulphathiazole (33% to 39%) and gentamicin (4.3% to 8.2%) increased significantly from 2015–2018 to 2019–2022 and resistance to chloramphenicol (10% to 8%) and cefotaxime (8% to 7%) decreased. Although the proportion of isolates predicted to be ciprofloxacin resistant remained unchanged in both the UK and the USA, the proportion of isolates predicted to show intermediate resistance to ciprofloxacin significantly decreased in the UK (from 9% to 8%) and increased in the USA (2% to 3%) from 2015–2018 to 2019–2022. We also compared the predicted resistance to individual antibiotics for isolates from the UK and the USA in 2019–2022 (Figure 1). The UK isolates had significantly higher predicted resistance to eight antibiotics, namely streptomycin (higher by 23%), ampicillin (by 25%), chloramphenicol (by 8%), trimethoprim (by 8%), trimethoprim-sulphathiazole (by 7%), kanamycin (by 1.3%), azithromycin (by 0.5%), and colistin (by 0.3%), and the USA had higher resistance to only two antibiotics, cefotaxime (higher by 5%) and gentamicin (by 5%). Resistance to ciprofloxacin was not significantly different between UK and USA, but significantly more isolates from the UK were predicted to have intermediate resistance (by 4%).

### Predicted resistance in MGT1 (MLST) STs

**Figure 2:**
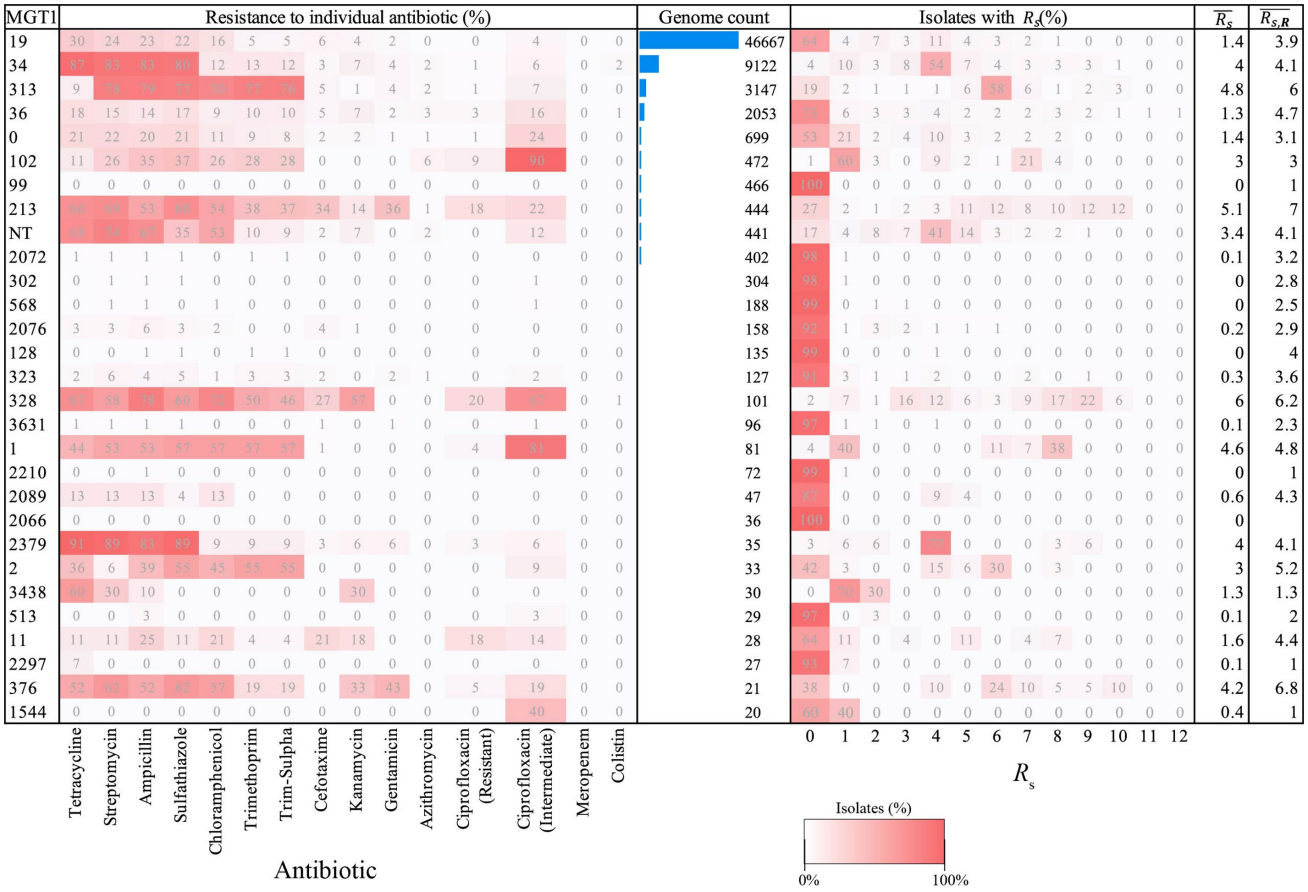
Predicted resistance to 14 antibiotics and aggregate resistance metrics for MGT1 STs. Only STs with ≥20 isolates are shown and STs are listed in descending order of the number of genomes that they include. A value of ‘0’ represents a new ST, while ‘NT’ (Not Typable) indicates that an ST could not be assigned (for example, due to missing data). The resistance score ($R_{S}$) for each isolate represents the number of antibiotics to which it is predicted to be resistant, and includes intermediate resistance to ciprofloxacin. The heatmap ‘Isolates with $R_{s}$(%)’ shows the distribution of isolates with a given resistant score within an MGT1 ST. ‘$\bar{R_{s}}$’ indicates the mean resistance score of all isolates within the MGT1 ST, and ‘$\bar{R_{s,R}}$’ indicates the mean resistance score of isolates excluding those predicted to be susceptible to all 14 antibiotics.

MGT1 (MLST) ST19 was the most frequent ST (71% isolates) followed by ST34 (14%), ST313 (5%) and ST36 (3%) (Figure 2; Supplementary Table S4). Most ST19 isolates were predicted to be susceptible to all antibiotics (64%), whereas most ST34 isolates were predicted to be resistant (intermediate/resistant for ciprofloxacin) to at least one antibiotic (96%). Similarly, ST313 isolates were predominantly resistant (intermediate/resistant for ciprofloxacin) to at least one antibiotic (81%), whereas ST36 isolates were predominantly susceptible to all antibiotics (73%).

Comparison of the mean resistance score of isolates in these STs revealed the following order: ST313 ($\bar{R_{s}}$= 4.8) > ST34 ($\bar{R_{s}}$= 4) > other STs ($\bar{R_{s}}$= 1.7) and ST19 ($\bar{R_{s}}$= 1.4) > ST36 ($\bar{R_{s}}$= 1.3). The mean resistance score of isolates with resistance to at least one antibiotic (including intermediate/resistant for ciprofloxacin) was highest for ST313 ($\bar{R_{s,R}}$= 6) followed by ST36 ($\bar{R_{s,R}}$= 4.7), then by other STs ($\bar{R_{s,R}}$= 4.2). Resistance in ST34 isolates ($\bar{R_{s,R}}$= 4.1) was assessed as significantly less than that in ST313 isolates, but similar to isolates in ST36 and other STs. Lastly, resistance in ST19 isolates ($\bar{R_{s,R}}$= 3.9) was significantly lower than that in ST313, ST34 and ST36, but similar to resistance in other STs. Resistance to individual antibiotics in MGT1 STs is compared in Supplementary Results. Additionally, the distribution of the well-known ASSuT-associated AMR genes in MGT1 ST34, and ACSSuT-associated AMR genes in MGT3 ST11 (or DT104) are also presented in Supplementary Results.

### AMR in higher level MGT STs

Analysis of MGT1 (MLST) STs gives a broad overview of AMR in the dataset, especially in the context of the most frequent STs, ST19 and ST34. To obtain a finer-grade understanding of relationships between genomic types and AMR, we classified isolates into higher-resolution MGT STs, with each isolate assigned to nine STs, MGT1-MGT9, where possible. We then used this classification to identify MGT STs in which a high proportion of isolates are predicted to be resistant to a particular antibiotic, or intermediate/resistant in the case of ciprofloxacin. For each antibiotic, we defined a “resistant MGT ST” as an MGT ST where at least 80% of isolates were predicted to be resistant to that antibiotic (see Supplementary Results for details of why the 80% cutoff was chosen and Supplementary Figure S7 for explanation of the process). For ciprofloxacin, we similarly defined a “resistant/intermediate MGT ST.” For any given antibiotic, MGT STs defined as resistant or ciprofloxacin intermediate/resistant consist of mutually exclusive sets of isolates.

In the complete dataset, we identified 407 MGT STs defined as resistant to at least one antibiotic, or resistant/intermediate to ciprofloxacin (Supplementary Table S5; Table 3; Supplementary Dataset 1). MGT STs defined as resistant/intermediate for ciprofloxacin or as resistant for all antibiotics except meropenem were identified (Table 3; Supplementary Figure S9), and spanned all levels, MGT1-MGT9 (Table 3). The majority of MGT STs defined as resistant (or resistant/intermediate for ciprofloxacin) were resistant to one antibiotic (146 of 407), with two MGT STs resistant or resistant/intermediate to nine antibiotics (Supplementary Table S5). We also applied a similar approach, i.e., using higher level MGT STs, to assess whether MGT STs with a high proportion of isolates (≥80%) associated with the ASSuT AMR genes [*bla*_TEM-1_, *strAB*, *sul2*, *tet*(B)] could be identified. However, higher level MGT STs were not deemed suitable for detecting this specific gene set (Supplementary Results).

**Table 3:** Number of MGT STs defined as resistant at each MGT level for each antibiotic.

Drug	Number of resistant isolates			Number of resistant MGT STs^a^									Total	Number of resistant isolates in resistant MGT STs		
	Complete dataset	2015–2022	2021–2022	MGT1	MGT2	MGT3	MGT4	MGT5	MGT6	MGT7	MGT8	MGT9		Complete dataset	2015–2022	2021–2022
Tetracycline	23,517	11,868	2,995	2	17	68	34	28	16	4	0	0	169	22,249	11,657	2,981
Streptomycin	22,885	9,684	2,327	4	20	66	26	19	20	3	0	0	158	21,508	9,555	2,264
Ampicillin	22,343	9,221	2,107	5	21	59	38	25	13	4	1	0	166	21,569	9,411	2078
Sulfathiazole	21,710	10,297	2,609	4	15	55	35	22	22	4	0	1	158	19,699	10,111	2,589
Chloramphenicol	11,876	3,527	886	0	16	41	42	35	33	10	0	0	177	7,903	2082	617
Trimethoprim	6,851	2060	475	1	4	22	18	5	8	5	0	0	63	4,116	782	229
Trim-Sulpha	6,452	1836	436	1	3	20	17	3	8	4	0	0	56	3,915	645	221
Cefotaxime	3,447	1,223	336	0	2	11	13	19	10	5	0	0	60	1,674	594	176
Kanamycin	2,958	1,159	343	0	3	8	8	5	5	3	0	0	32	1,162	360	155
Gentamicin	1856	1,036	395	0	2	6	5	8	2	1	0	0	24	627	382	211
Azithromycin	629	290	45	0	0	0	2	1	0	0	0	0	3	81	15	0
Ciprofloxacin^b^	4,476	1842	372	2	5	15	13	11	6	1	0	0	53	1904	592	99
Colistin	219	120	10	0	0	0	0	1	0	0	0	0	1	15	14	0
Meropenem	14	12	7	0	0	0	0	0	0	0	0	0	0	0	0	0

^a^A resistant MGT ST is an MGT ST where at least 80% of isolates are resistant. ^b^Intermediate resistance is also included for this antibiotic.

### Resistance to prescribed and last-line antibiotics

Our analysis focused on two antibiotics commonly prescribed to treat human STm infections, namely ciprofloxacin and cefotaxime, particularly in relation to recent (2021–2022) isolates. Resistance to azithromycin, a third common treatment, and two last-line antibiotics meropenem (carbapenem) and colistin, was also examined, but as this was observed only rarely and sporadically, these data are provided in Supplementary Results.

### Cefotaxime

We identified 3,446 (46%) isolates predicted to be resistant to cefotaxime, with 1,578 (46%) of these assigned to 60 MGT STs defined as cefotaxime-resistant (Supplementary Dataset 1). These MGT STs also included 96 isolates predicted to be susceptible (Supplementary Figure S9). Year of isolation was available for 923 (55%) isolates in these 60 MGT STs. Using these metadata, 22 MGT STs were identified as major STs (≥10 isolates) in 2015–2022, with MGT3 ST171 including the most isolates (*n =* 60) and the remaining MGT STs having a mean of 21 isolates each (Supplementary Dataset 1).

**Figure 3:**
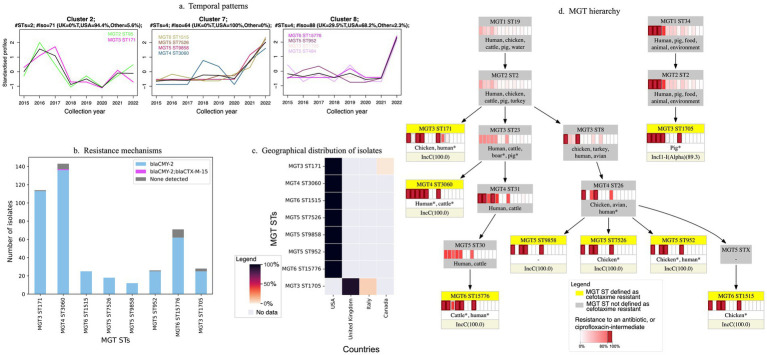
Characteristics of eight major MGT STs in 2021–2022 defined as cefotaxime resistant. A resistant MGT ST is an MGT ST where at least 80% of isolates are predicted to be resistant. **(a)** Three temporal patterns. Three of eight distinct temporal clusters identified for cefotaxime-resistant MGT STs in 2015–2022 (Supplementary Figure S12) contained the eight major 2021–2022 MGT STs (> = 10 isolates) (MGT3 ST171, MGT4 ST3060, MGT6 ST1515, MGT5 ST7526, MGT5 ST9858, MGT5 ST952, MGT6 ST15776, MGT3 ST1705). MGT2 ST95 and MGT5 ST484 in these clusters were not major STs in 2021–2022 and are shown in part **(a)** only. MGT3 ST171 (Cluster 2) exhibited a peak in 2017, with the MGT STs in Clusters 7 and 8 (MGT5 ST952, MGT6 ST15776, MGT3 ST1705) peaking in 2022. MGT STs in Cluster 7 showed a gradual increase from 2021, while MGT STs in Cluster 8 sharply increased in 2022. **(b)** Resistance mechanisms in the eight major MGT STs. *bla*_CMY-2_ is present in most or all isolates, while a few isolates in MGT4 ST3060 also carry *bla*_CTX-M-15_. **(c)** Geographic distribution of isolates in the eight MGT STs. **(d)** MGT hierarchy and AMR profiles. The eight resistant MGT STs (highlighted in yellow) are shown in the context of their lower MGT-level STs (in grey). Lower MGT levels encompass broader genomic diversity, forming a natural tree-like hierarchy, while higher MGT levels capture finer genomic resolution by grouping closely related isolates. Since MGT levels 1 to 7 are based on mutually exclusive genomic schemes, a higher-level MGT ST may comprise isolates from multiple lower-level MGT STs. ‘MGT5 STX’ indicates that ≥50% of MGT6 ST1515 isolates could not be assigned to a single MGT5 ST. The heatmap for each MGT ST shows the percentage of isolates resistant to (from left to right) tetracycline, streptomycin, ampicillin, sulfathiazole, chloramphenicol, trimethoprim, trimethoprim-sulfathiazole, cefotaxime, kanamycin, gentamicin, azithromycin, ciprofloxacin (resistant and intermediate), meropenem or colistin. Dark red boxes indicate that the MGT ST is defined as a resistant MGT ST for that antibiotic. The five most frequent isolation sources for each ST are also listed, with an asterisk (*) indicating sources with <10 isolates. The last row presents the likely plasmid replicon associated with *bla*_CMY-2_ carriage for each ST, with the percentage of isolates carrying this replicon type indicated in brackets.

Temporal clustering of the 22 major MGT STs defined as cefotaxime-resistant in 2015–2022 revealed eight distinct patterns (clusters) (Supplementary Figure S12; Figure 3a). To facilitate inter-MGT ST comparison, isolate counts for each ST were normalized to the mean annual count for that ST, with temporal variations in isolate numbers represented as fold-changes relative to the mean. As an example, MGT4 ST3060 exhibited an approximately 1-fold increase in 2018, falling below the mean in 2020, and then increasing to approximately 2-fold in 2022. Clusters were numbered in order of the earliest initial peak among the constituent STs. For example, Cluster 1 contains MGT STs that were already present in 2015, whereas Cluster 8 contains MGT STs that appeared in 2022. Seven clusters were restricted to a single geographical region: five contained isolates predominantly from the USA (Clusters 2, 4, 5, 7, and 8), one was limited to the UK (Cluster 1), and another to other regions (Cluster 6), with MGT3 ST136 comprising isolates from Mexico and MGT5 ST5868 from China. In contrast, Cluster 3 contained isolates from multiple countries.

In 2021–2022, the most recent years for which data were included, eight MGT STs defined as cefotaxime-resistant are classified as major STs (≥10 isolates) (Figure 3). Of these, seven are predominantly composed of isolates from the USA (Figure 3c) and belong to MGT1 ST19 (Figure 3d). In contrast MGT3 ST1705, which is primarily composed of isolates from the UK and Italy (Figure 3c), is part of MGT1 ST34 (Figure 3d).

Source metadata is available for only a subset of isolates (Figure 3d). Among these, the UK-associated MGT3 ST1705 included a small number of isolates from pigs. The USA-associated MGT STs contained a limited number of human isolates, while the remaining isolates were primarily linked to either poultry (MGT3 ST171, MGT5 ST7526, MGT5 ST952, MGT6 ST1515) or cattle (MGT6 ST15776, MGT4 ST3060). One ST, MGT5 ST9858, lacked source metadata. However, when considering these MGT STs in the context of their lower-level MGT classifications (Figure 3d), a predominantly non-human source could be identified. Specifically, MGT3 ST171 and MGT3 ST8 were largely associated with avian sources, while MGT3 ST23 was primarily linked to cattle and pigs. Further differentiation of MGT3 ST23 at the MGT4 level indicated a more specific non-human origin, with MGT4 ST3060 and MGT4 ST31 consisting exclusively of cattle isolates.

Cefotaxime resistance in these 60 MGT STs was conferred by eight distinct genes or gene combinations (Supplementary Figure S14a). *bla*_CMY-2_ was the most common, found in 49 MGT STs, including the eight major MGT STs in 2021–2022 (Figure 3b). Another 11 MGT STs contained genes such as *bla*_CTX-M-9_, *bla*_CTX-M-15_ and *bla*_SHV-2A_. As these genes have been reported to be carried on plasmids in *Enterobacteriaceae* (Carattoli, 2009; Rozwandowicz et al., 2018), plasmid replicon types identified by PlasmidFinder were examined, even though these cannot usually be directly linked to AMR genes from short-read data. Multiple plasmid replicon types were detected in the eight major MGT STs in 2021–2022. Of note, most MGT3 ST1705 isolates (UK) had an IncI1α replicon, while the remaining seven MGT STs (USA) had an IncC replicon (Figure 3d). Both these replicon types have been previously associated with the carriage of genes for cefotaxime resistance, (including *bla*_CMY-2_) (Foley et al., 2021; Martin et al., 2012).

As *bla*_CMY-2_ is the most common mechanism of cefotaxime resistance observed in resistant MGT STs in 2021–2022, we assessed the reliability of prediction of cefotaxime resistance based identification of this gene in WGS data and the subsequent designation of resistant MGT STs. When using our pipeline, we observed an underreporting of resistance predictions, i.e., where isolates were predicted to be susceptible by our pipeline, but predicted to be resistant using at least one other method (e.g., alternative assembly or assembly-free method). This was observed in at most 14 genomes (out of 437) representing an error rate of at most 3% (see Supplementary Results for further details). Specifically, among the 14 discordant predictions, the genomes of nine isolates were found to carry the complete *bla*_CMY-2_ gene, and five to carry either a partial *bla*_CMY-2_ (≤10% missing) or a *bla*_CMY-2_-like allele (99% identity). The nine genomes where the complete *bla*_CMY-2_ gene was identified using alternative methods would subsequently be predicted as resistant using abritAMR (Sherry et al., 2023). Thus, in these genomes, the discrepant predictions reflect discrepancies in the assembly of *bla*_CMY-2_. In the remaining five genomes, variations were present in the resistance gene that may alter phenotypic resistance, and the discrepant predictions here reflect the AMR tool’s (abritAMR) logic for identification and prediction. Nevertheless, an error rate of at most 3% is comparable to the overall accuracy of the resistance prediction by abritAMR (Sherry et al., 2023) and from our own validation (Supplementary Table S3). Additionally, in the identification of MGT STs defined as resistant, a buffer of 20% susceptible isolates is permitted, and the observed error rate of 3% is well below this range. Thus, we judged that predictions of MGT STs as resistant would be both reliable and robust.

Half of the isolates predicted to be cefotaxime resistant (54%, 1,868) did not belong to MGT STs defined as cefotaxime resistant (Supplementary Figure S9). These isolates were distributed across 24 MGT1 STs, 73 MGT2 STs and 298 MGT3 STs. These isolates were found in all locations: USA (936), UK (158) and Other (311), with the remaining missing country information. These isolates had 44 different complete resistance genes, incomplete resistance genes (≤10% missing sequence), or combinations of these, with *bla*_CMY-2_ being the most frequent, found in 74% of isolates (Supplementary Table S6).

### Ciprofloxacin

**Figure 4:**
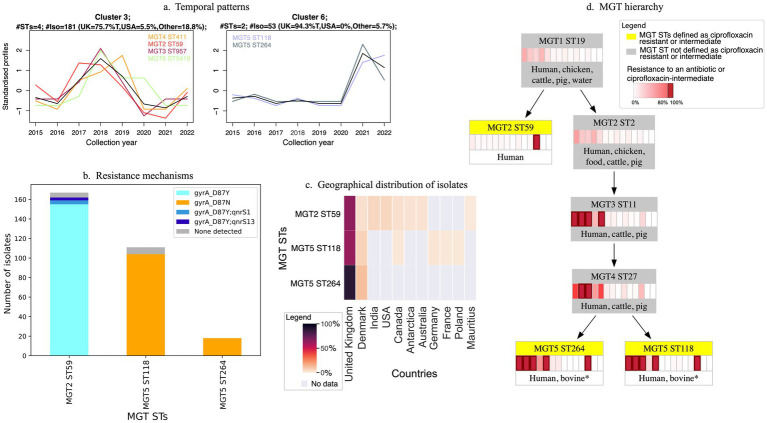
Characteristics of three major MGT STs defined as ciprofloxacin-intermediate in 2021–2022. A resistant MGT ST is an MGT ST where at least 80% of isolates are predicted to be resistant. **(a)** Two temporal patterns. Two of seven distinct temporal clusters identified for MGT STs defined as ciprofloxacin resistant or intermediate in 2015–2022 (Supplementary Figure S13) contained the three major 2021–2022 MGT STs. Three MGT STs (MGT4 ST411, MGT3 ST957, MGT6 ST5419) in these clusters were not major STs in 2021–2022 and are shown in part **(a)** only. MGT2 ST59 (Cluster 3) first peaked in 2017–2018 but reemerged in 2022, while MGT5 ST118 and MGT5 ST264 (Cluster 6) emerged more recently, peaking in 2021. **(b)** Resistance mechanisms in the three MGT STs. Most isolates in these three MGT STs harboured a single GyrA mutation (predicted to confer intermediate resistance), either D87N in MGT5 ST118 and MGT5 ST264, or D87Y in MGT2 ST59. A few MGT2 ST59 isolates also carried *qnrS1* (*n =* 4) or *qnrS13* (*n =* 3), which together with the GyrA mutation would predict full ciprofloxacin resistance. **(c)** Geographic distribution of isolates in the three MGT STs. **(d)** MGT hierarchy and AMR profiles. The three MGT STs defined as intermediate to ciprofloxacin (highlighted in yellow) are shown in the context of their lower MGT-level STs (in grey). The heatmap for each MGT ST shows the percentage of isolates resistant to (from left to right) tetracycline, streptomycin, ampicillin, sulfathiazole, chloramphenicol, trimethoprim, trimethoprim-sulfathiazole, cefotaxime, kanamycin, gentamicin, azithromycin, ciprofloxacin (resistant and intermediate), meropenem or colistin. Dark red boxes indicate that the MGT ST was defined as a resistant MGT ST for that antibiotic, or intermediate in the case of ciprofloxacin. The five most frequent isolation sources for each ST are listed, with an asterisk indicating sources with <10 isolates.

We identified 53 MGT STs that were predicted to be intermediate or resistant to ciprofloxacin. These MGT STs consisted of 165 of 485 (34%) resistant isolates, 1,675 of 3,991 (42%) intermediate isolates, and included 64 susceptible isolates (Supplementary Dataset 1 and Supplementary Figure S9). In these 53 MGT STs, 906 isolates (48%) had information about year of collection, and using these metadata 20 MGT STs were identified as major STs (≥10 isolates) in 2015–2022. Of these 20 MGT STs, MGT2 ST59 has the most isolates in this timeframe (*n =* 120) and the other 19 STs have a mean of 18 isolates each (Supplementary Dataset 1). Temporal clustering of these 20 major MGT STs revealed seven distinct patterns of temporal distribution (Supplementary Figure S13; Figure 4a). Five clusters (Clusters 1, 2, 3, 6 and 7) comprised isolates mostly from UK, and two clusters (Clusters 4 and 5) comprised isolates mostly from the Other set (from China, Mexico, Colombia, and Australia).

During 2021–2022, the most recent years for which data were included, only three MGT STs were classified as major STs: MGT2 ST59, MGT5 ST118, and MGT5 ST264 (Figure 4). While all three MGT STs comprised isolates predominantly from the UK, MGT2 ST59 was also detected in the USA and several other countries, including India, Denmark, Canada, and Australia (Figure 4c). Both MGT5 STs were also found in other European countries, such as Denmark. The MGT5 STs both fall within MGT3 ST11 (DT104) and all three MGT STs belong to MGT1 ST19 (Figure 4d). Although host information was unavailable for most isolates, all three STs included isolates identified as originating from human hosts and isolates from both MGT5 STs were also recovered from bovine sources (Figure 4d).

The 53 MGT STs defined as ciprofloxacin-resistant or intermediate were classified into 14 groups based on shared mutations or AMR genes (Supplementary Figure S15a and Supplementary Dataset 1). Among these, 18 MGT STs contained isolates that were predicted to exhibit complete phenotypic resistance (2–4 resistance mechanisms), with the proportion of resistant isolates in these STs ranging from 100% in MGT2 ST333 to 3.7% in MGT1 ST1 (Supplementary Figure S15d). Isolates in the remaining 35 MGT STs have only at most only one resistance mechanism, resulting in a prediction of intermediate resistance to ciprofloxacin (Supplementary Figure S15d). Isolates in 17/35 of these MGT STs had mutations, predominantly in *gyrA*, with isolates in one ST having a *gyrB* mutation (Supplementary Dataset 1). For 14 of these 17 MGT STs, 100% of isolates carry a mutation, while for the remaining three MGT STs 95–96% of isolates have a mutation. The other 18/35 MGT STs are defined as intermediate because they carry a ciprofloxacin resistance gene. In 10 of these MGT STs all isolates have a resistance gene [e.g., *qnrS1*, *qnrB2* or *aac(6′)-Ib-cr*; Supplementary Dataset 1] while in the remaining eight, only 80–98% of isolates do. Notably, among the MGT STs containing isolates from 2021 to 2022 (Figure 4b), MGT5 ST118 and MGT5 ST264 were predicted to have intermediate resistance to ciprofloxacin due to a single mutation in *gyrA*, resulting in the D87N mutation in GyrA. MGT2 ST59 isolates were predicted to be predominantly intermediate, with all isolates carrying a *gyrA* mutation giving D87Y, but 4% also carry *qnrS1* or *qnrS13*, combinations predicted to confer full resistance. As mutations in *gyrA* are the most common ciprofloxacin resistance mechanism observed in intermediate MGT STs in 2021–2022, we assessed the reliability of predicting MGT STs defined as intermediate based on these mutations. We judged that such predictions would be reliable, as *gyrA* is a core gene of *Salmonella* (MGT8) (Payne et al., 2020). Since *gyrA* is a core gene, it can be reliably identified and genotyped. Additionally, any mutations, including those that confer resistance to ciprofloxacin, are reliably identifiable (Payne et al., 2020). Further details can be found in the Supplementary Results.

The majority of isolates predicted to be ciprofloxacin resistant (66%, 320) or intermediate (58%, 2,316) did not belong to MGT STs defined as ciprofloxacin resistant or intermediate (Supplementary Figure S9). These resistant or intermediate isolates were highly genomically diverse, distributed across 36 different MGT1 STs, 91 MGT2 STs, and 453 MGT3 STs. These isolates were found in all locations: UK (797), USA (399) and Other (649) with the remaining lacking country information. Collectively, these isolates had 104 distinct ciprofloxacin resistance determinants, including genes, incomplete genes (with ≤10% missing sequence), mutations, or combinations of these (Supplementary Table S7).

## Discussion

In this study, we analyzed the predicted resistance profiles of ~65,000 publicly available STm genomes for 14 antibiotics, including clinically relevant ones. The dataset encompassed genomic data from multiple countries, incorporating high-quality genomic surveillance data from the UK (from 2015 onwards) and the USA (from 2019 onwards). In the complete dataset, nearly half of the isolates were predicted to be resistant to 1–12 antibiotics, with mean resistance to four antibiotics per isolate. However, predicted resistance varied by geographic location, time period and by MGT1 ST. From 2015–2022, the proportion of resistant isolates was significantly higher in the UK (60%) compared to the USA (42%). The proportion of resistant isolates in the UK significantly decreased from 2015–2018 to 2019–2022, whereas in the USA, this increased over the same period. Despite these shifts, the mean resistance score among resistant isolates (or intermediate isolates for ciprofloxacin) remained stable in both regions, suggesting that these changes were driven by fluctuations in the number of resistant isolates rather than by widespread acquisition or loss of resistance to additional antibiotic classes.

Resistance to individual antibiotics, however, varied across locations, timeframes, and MGT1 STs, with fluctuations in the proportion of resistant isolates. Additionally, previously established chromosomal resistance patterns associated with traditional phenotypic resistance profiles, such as ASSuT in ST34 and ACSSuT in DT104, were not well preserved (see Supplementary Results). These findings highlight the highly dynamic nature of AMR in STm.

Several factors may have contributed to the observed changes in predicted resistance. Some examples include proximity to other species with transferable AMR genes from which they could be acquired (Baker et al., 2024), changing selection pressures, such as altered AMR exposure for different STm populations (Mellor et al., 2019), and co-selection (Murray et al., 2024). Furthermore, sampling strategies can also influence observed resistance trends. For instance, from 2015 to 2022, surveillance in the UK was part of a national framework aimed to sequence all *Salmonella* isolates. As a result, the observed decline in resistance over this period in the UK likely reflects a genuine reduction, potentially driven by the implementation of antimicrobial stewardship programs (Johnson et al., 2015; Helliwell et al., 2020). Although stewardship recommendations also exist in the USA (Shrestha et al., 2023; Ruegg, 2022), the apparent significant increase in resistance among USA isolates may be partly attributable to the implementation of a national *Salmonella* surveillance program in 2019, which may have introduced more comprehensive and less biased sampling. The dynamic AMR landscape highlights the importance of implementing continuous and standardized monitoring strategies to track resistance trends effectively.

Once monitoring systems are established, the next steps involve tracking strains and their AMR gene content systematically (World Health Organization, 2023), which can be effectively achieved using MGT. The MGT typing system, which assigns nine levels of STs per isolate, provides a scalable, standardized, and multi-resolution typing framework for STm. As demonstrated here, we utilized MGT to stratify the dataset into higher-resolution STs, enabling the identification of STs with high proportions (≥80%) of isolates resistant to specific antibiotics (or resistant/intermediate to ciprofloxacin). Across 14 antibiotics, we identified 407 MGT STs defined as resistant (or ciprofloxacin-intermediate), spanning all MGT levels. In particular, this approach presents a way to correlate elements of the core genome, via sequence typing (i.e., MGT STs), with the accessory genome (Croll and McDonald, 2012; Segerman, 2012) (e.g., AMR determinants that are horizontally acquired), such that the clones or strains carrying AMR genes/mutations can be easily identified, the standardized identity (i.e., the MGT ST) can be shared, and subsequently monitored. The utility of such a correlation is that an additional layer of confidence is added to the predicted resistance. For example, the way that MGT STs are defined as resistant (or ciprofloxacin-intermediate) gives a 20% buffer of susceptible isolates, which enables MGT STs with resistance (or intermediate resistance for ciprofloxacin) to be inferred, even in erroneous absence of resistance mechanisms (such as low sequence quality or assembly errors) or real sporadic loss of resistance (as such plasmid instability). This was exemplified in the potential underreporting of cefotaxime resistance in 14 of 437 isolates, representing an error rate of at most 3%, well within the 20% buffer. This built-in tolerance ensures robust resistance classification, mitigating the impact of technical errors while maintaining high specificity in resistance tracking.

MGT provides a naturally hierarchical setting to study related isolates (Figures 3d, 4d) where higher-resolution STs can be analyzed within their broader lower-resolution counterparts. This approach enables epidemiological inferences, particularly when metadata are incomplete, allowing us to track isolates over time, and by geographic spread, and by host association. Such inferences are described below for MGT STs defined as cefotaxime resistant, and ciprofloxacin-resistant or intermediate.

In the last two years examined here, 2021–2022, eight MGT STs were defined as cefotaxime resistant (MGT3 ST1705, MGT3 ST171, MGT4 ST3060, MGT6 ST15776, MGT5 ST9858, MGT5 ST7562, MGT5 ST952, MGT6 ST1515). These eight MGT STs can be grouped into three distinct sets. MGT3 ST1705 was the only ST predominantly found in the UK and the only one associated with pigs. Most isolates also carry a distinct plasmid replicon type (IncI1α). The remaining seven STs are from the USA, associated with either poultry or cattle, and commonly carry an IncC plasmid replicon. Both IncI1α and IncC plasmids have been previously linked to *bla*_CMY-2_-mediated cefotaxime resistance (Foley et al., 2021; Folster et al., 2014). The association of distinct plasmid replicon types with distinct MGT STs suggests that resistance to cefotaxime was independently acquired by each group. Notably, AMR profiles further distinguished the poultry- and cattle-associated STs. Poultry-associated STs exhibited high predicted resistance to tetracycline and sulfathiazole, while cattle-associated STs showed additional predicted resistance to streptomycin and chloramphenicol. This differentiation highlights the ability of our approach to identify and track resistant MGT STs linked to distinct reservoirs. Overall, this study demonstrates the power of genomics-based tracking in monitoring the spread of highly resistant strains, offering precise surveillance of cefotaxime-resistant STs across different hosts and regions.

In the final two years examined here (2021–2022), one MGT2 ST (MGT2 ST59) and two MGT5 STs (MGT5 ST118 and MGT5 ST264) were defined as ciprofloxacin resistant (presence of ≥ two resistance mechanisms) or intermediate (presence of one resistance mechanism). Within the MGT hierarchy, both MGT5 STs belong to the same lower-level MGT ST, and all three MGT STs defined as ciprofloxacin-resistant or intermediate are part of the same MGT1 ST. Lower-level MGT STs (e.g., MGT1 ST19) group isolates more broadly, resulting in lower resolution and generally larger sets of isolates. Consequently, the percentage of isolates predicted to be resistant or intermediate at these levels may be quite low. In contrast, higher-resolution MGT levels (e.g., MGT2 ST59, MGT5 ST118 and MGT5 ST264) enable finer differentiation based on genomic similarity. As demonstrated in this study, these higher-level MGT STs can be used to track specific features such as AMR. This highlights the utility of the multilevel typing approach, which enables users to select the most appropriate resolution level for representing and analysing their isolates.

We clustered MGT STs defined as resistant (or ciprofloxacin-intermediate) using two approaches: (i) temporal clustering, based on the annual count of isolates (Figures 3a, 4a, and Supplementary Figures S12, S13), and (ii) clustering by AMR mechanisms, based on genetic similarity (Supplementary Figure S14a, S15a). Temporal clustering allowed us to identify dominant trends over time. Similarly clustering by AMR mechanism also enabled the detection of predominant genetic determinants of AMR within the dataset. For example, although several mechanisms of resistance to cefotaxime were seen in earlier isolates (Supplementary Figure S14a), only one mechanism, *bla*_CMY-2_, was observed in the last two years examined (Figure 3b). Although the application of unsupervised clustering approaches as used here are common in other life-sciences domains [such as time series proteomic measurements (Kaur et al., 2020)], to our knowledge their application to epidemiological data, by considering MGT STs as multivariate, is novel. These approaches provided a natural setting to understand the trends of AMR in a dataset and enabled comparison between different MGT STs with varying abundances. Thus, in this work we present an approach for applying clustering methods for epidemiological surveillance of resistant (or ciprofloxacin-intermediate) isolates. Importantly, while clustering by AMR mechanisms relies on the extraction of information from the genome sequence, temporal clustering relies on the availability of information about the time of isolation. Thus, the interpretation of an observed temporal trend depends on both information about year of isolation (as used in this study) and the underlying sampling quality. While the importance of metadata is well known (Cernava et al., 2022), equal attention must be given to the importance of sampling (Baker et al., 2023). When sampling is unbiased and conducted consistently over time, the observed trends in the prevalence of specific MGT STs can be considered reliable indicators of their true epidemiology. This highlights the critical importance of systematic sampling in generating meaningful epidemiological insights, including for accurately tracking the emergence, persistence, and spread of AMR.

Lastly, the AMR data generated and analysed for the set of isolates in this study are available in MGTdb (see Footnote 1). In future work, we will expand the functionality of this platform to enable automated integration and analysis of AMR for all isolates in the database, including for new isolates added to the database, automatic routine computation and compilation of high risk MGT STs and other analysis capabilities (e.g., generalized AMR trends). These enhancements will aim to facilitate scalable and routine application of the framework demonstrated in this study, supporting real-time genomic surveillance of antimicrobial resistance.

## Conclusion

In this study, we provide a comprehensive overview of predicted AMR in publicly available global STm genome sequence data. In particular, we demonstrate the utility of integrating MGT to identify and enable precise tracking of resistant MGT STs and temporal and spatial clustering to identify trends of MGT STs with high levels of resistance. The approaches presented here can be effectively applied to standardized global comprehensive surveillance of AMR in STm, providing opportunities for early detection, data-driven decision making, targeted interventions, and measurements of effectiveness of interventions, and should be applicable to other organisms.

## Data Availability

The original contributions presented in the study are included in the article/Supplementary material, further inquiries can be directed to the corresponding author.

## Footnotes

^1^ https://mgtdb.unsw.edu.au/typhimurium

## References

[ref1] Abdi H. (2007). Binomial distribution: binomial and sign tests. Encyclopedia Measurement Stat. 22, 1–12. doi: 10.4135/9781412952644

[ref2] Achtman M. Wain J. Weill F.-X. Nair S. Zhou Z. Sangal V. . (2012). Multilocus sequence typing as a replacement for serotyping in *Salmonella enterica*. PLoS Pathog. 8:e1002776. doi: 10.1371/journal.ppat.1002776, PMID: 22737074 PMC3380943

[ref3] Ashton P. M. Nair S. Peters T. M. Bale J. A. Powell D. G. Painset A. . (2016). Identification of *Salmonella* for public health surveillance using whole genome sequencing. PeerJ. 4:e1752. doi: 10.7717/peerj.1752, PMID: 27069781 PMC4824889

[ref4] Baker K. S. Jauneikaite E. Hopkins K. L. Lo S. W. Sánchez-Busó L. Getino M. . (2023). Genomics for public health and international surveillance of antimicrobial resistance. Lancet Microbe. 4, e1047–e1055. doi: 10.1016/S2666-5247(23)00283-5, PMID: 37977162

[ref5] Baker S. Thomson N. Weill F.-X. Holt K. E. (2018). Genomic insights into the emergence and spread of antimicrobial-resistant bacterial pathogens. Science 360, 733–738. doi: 10.1126/science.aar3777, PMID: 29773743 PMC6510332

[ref6] Baker M. Zhang X. Maciel-Guerra A. Babaarslan K. Dong Y. Wang W. . (2024). Convergence of resistance and evolutionary responses in *Escherichia coli* and *Salmonella enterica* co-inhabiting chicken farms in China. Nat. Commun. 15:206. doi: 10.1038/s41467-023-44272-1, PMID: 38182559 PMC10770378

[ref7] Bawn M. Alikhan N.-F. Thilliez G. Kirkwood M. Wheeler N. E. Petrovska L. . (2020). Evolution of *Salmonella enterica* serotype Typhimurium driven by anthropogenic selection and niche adaptation. PLoS Genet. 16:e1008850. doi: 10.1371/journal.pgen.1008850, PMID: 32511244 PMC7302871

[ref8] Besser T. Goldoft M. Pritchett L. Khakhria R. Hancock D. Rice D. . (2000). Multiresistant *Salmonella* Typhimurium DT104 infections of humans and domestic animals in the Pacific northwest of the United States. Epidemiol. Infect. 124, 193–200. doi: 10.1017/S0950268899003283, PMID: 10813142 PMC2810900

[ref9] Branchu P. Bawn M. Kingsley R. A. (2018). Genome variation and molecular epidemiology of *Salmonella enterica* serovar Typhimurium pathovariants. Infect. Immun. 86, 79–118. doi: 10.1128/iai.00079-18 PMC6056856 29784861

[ref10] Briggs C. E. Fratamico P. M. (1999). Molecular characterization of an antibiotic resistance gene cluster of *Salmonella* Typhimurium DT104. Antimicrob. Agents Chemother. 43, 846–849. doi: 10.1128/AAC.43.4.846, PMID: 10103189 PMC89215

[ref11] Carattoli A. (2009). Resistance plasmid families in *Enterobacteriaceae*. Antimicrob. Agents Chemother. 53, 2227–2238. doi: 10.1128/AAC.01707-08, PMID: 19307361 PMC2687249

[ref12] Carattoli A. Zankari E. Garcìa-Fernandez A. Larsen M. V. Lund O. Villa L. . (2014). *In silico* detection and typing of plasmids using PlasmidFinder and plasmid multilocus sequence typing. Antimicrob. Agents Chemother. 58, 3895–3903. doi: 10.1128/AAC.02412-14, PMID: 24777092 PMC4068535

[ref13] Centers for Disease Control and Prevention. (2022). U.S. Department of Health & human services. Whole genome sequencing 2022. Available online at: https://www.cdc.gov/pulsenet/php/wgs/ (accessed August 15, 2022).

[ref14] Cernava T. Rybakova D. Buscot F. Clavel T. McHardy A. C. Meyer F. . (2022). Metadata harmonization–standards are the key for a better usage of omics data for integrative microbiome analysis. Environ. Microbiome 17:33. doi: 10.1186/s40793-022-00425-1, PMID: 35751093 PMC9233336

[ref15] Cochrane G. Karsch-Mizrachi I. Takagi T. International Nucleotide Sequence Database Collaboration (2016). The international nucleotide sequence database collaboration. Nucleic Acids Res. 44, D48–D50. doi: 10.1093/nar/gkv1323, PMID: 26657633 PMC4702924

[ref16] Conley Z. C. Bodine T. J. Chou A. Zechiedrich L. (2018). Wicked: the untold story of ciprofloxacin. PLoS Pathog. 14:e1006805. doi: 10.1371/journal.ppat.1006805, PMID: 29494701 PMC5832386

[ref17] Croll D. McDonald B. A. (2012). The accessory genome as a cradle for adaptive evolution in pathogens. PLoS Pathog. 8:e1002608. doi: 10.1371/journal.ppat.1002608, PMID: 22570606 PMC3343108

[ref18] Department of Health and Aged Care, Australian Government. (2023). National Notifiable Diseases Surveillance System (NNDSS) public dataset – *Salmonella*. Available online at: https://www.health.gov.au/resources/publications/national-notifiable-diseases-surveillance-system-nndss-public-dataset-salmonella?language=en (accessed September 5, 2023).

[ref19] Ehuwa O. Jaiswal A. K. Jaiswal S. (2021). *Salmonella*, food safety and food handling practices. Foods. 10:907. doi: 10.3390/foods10050907 33919142 PMC8143179

[ref20] Fierer J. (2022). Invasive non-typhoidal *Salmonella* (iNTS) infections. Clin. Infect. Dis. 75, 732–738. doi: 10.1093/cid/ciac035 35041743

[ref21] Foley S. L. Kaldhone P. R. Ricke S. C. Han J. (2021). Incompatibility group I1 (IncI1) plasmids: their genetics, biology, and public health relevance. Microbiol. Mol. Biol. Rev. 85, 10–1128. doi: 10.1128/MMBR.00031-20 PMC8139525 33910982

[ref22] Folster J. P. Tolar B. Pecic G. Sheehan D. Rickert R. Hise K. . (2014). Characterization of *bla*_CMY_ plasmids and their possible role in source attribution of *Salmonella enterica* serotype Typhimurium infections. Foodborne Pathog. Dis. 11, 301–306. doi: 10.1089/fpd.2013.1670 24484290 PMC4620657

[ref23] Foodborne Disease Burden Epidemiology Reference Group (FERG), Monitoring and Surveillance Nutrition and Food Safety (MNF), Nutrition and Food Safety (NFS) (2023). Whole genome sequencing as a tool to strengthen foodborne disease surveillance and response: Module 3: Whole genome sequencing in foodborne disease routine surveillance. Geneva: Foodborne Disease Burden Epidemiology Reference Group (FERG), Monitoring and Surveillance Nutrition and Food Safety (MNF), Nutrition and Food Safety (NFS).

[ref24] Gansner E. R. (2009). Drawing graphs with Graphviz. technical report. Murray Hill, NJ: AT&T Bell Laboratories, Murray, Tech Rep, Tech Rep.

[ref25] Gymoese P. Sørensen G. Litrup E. Olsen J. E. Nielsen E. M. Torpdahl M. (2017). Investigation of outbreaks of *Salmonella enterica* serovar Typhimurium and its monophasic variants using whole-genome sequencing, Denmark. Emerg. Infect. Dis. 23, 1631–1639. doi: 10.3201/eid2310.161248, PMID: 28930002 PMC5621559

[ref26] Helliwell R. Morris C. Raman S. (2020). Antibiotic stewardship and its implications for agricultural animal-human relationships: insights from an intensive dairy farm in England. J. Rural. Stud. 78, 447–456. doi: 10.1016/j.jrurstud.2020.07.008

[ref27] Johnson A. P. Ashiru-Oredope D. Beech E. (2015). Antibiotic stewardship initiatives as part of the UK 5-year antimicrobial resistance strategy. Antibiotics. 4, 467–479. doi: 10.3390/antibiotics4040467, PMID: 27025636 PMC4790308

[ref28] Kaur S. Payne M. Luo L. Octavia S. Tanaka M. M. Sintchenko V. . (2022). MGTdb: a web service and database for studying the global and local genomic epidemiology of bacterial pathogens. Database 2022:baac094. doi: 10.1093/database/baac094, PMID: 36367311 PMC9650772

[ref29] Kaur S. Peters T. J. Yang P. Luu L. D. W. Vuong J. Krycer J. R. . (2020). Temporal ordering of omics and multiomic events inferred from time-series data. NPJ Syst Biol Appl. 6:22. doi: 10.1038/s41540-020-0141-0, PMID: 32678105 PMC7366653

[ref30] Krause K. M. Serio A. W. Kane T. R. Connolly L. E. (2016). Aminoglycosides: an overview. Cold Spring Harb. Perspect. Med. 6:a027029. doi: 10.1101/cshperspect.a027029, PMID: 27252397 PMC4888811

[ref31] Kumar L. Futschik M. E. (2007). Mfuzz: a software package for soft clustering of microarray data. Bioinformation 2, 5–7. doi: 10.6026/97320630002005, PMID: 18084642 PMC2139991

[ref32] Li J. Zhang H. Ning J. Sajid A. Cheng G. Yuan Z. . (2019). The nature and epidemiology of OqxAB, a multidrug efflux pump. Antimicrob. Resist. Infect. Control 8, 1–13. doi: 10.1186/s13756-019-0489-3 30834112 PMC6387526

[ref33] Lucarelli C. Dionisi A. M. Filetici E. Owczarek S. Luzzi I. Villa L. (2012). Nucleotide sequence of the chromosomal region conferring multidrug resistance (R-type ASSuT) in *Salmonella* Typhimurium and monophasic *Salmonella* Typhimurium strains. J. Antimicrob. Chemother. 67, 111–114. doi: 10.1093/jac/dkr391, PMID: 21990047

[ref34] Martin L. C. Weir E. K. Poppe C. Reid-Smith R. J. Boerlin P. (2012). Characterization of *bla*_CMY-2_ plasmids in *Salmonella* and *Escherichia coli* isolates from food animals in Canada. Appl. Environ. Microbiol. 78, 1285–1287. doi: 10.1128/AEM.06498-11, PMID: 22156427 PMC3273016

[ref35] McKnight P. E. Najab J. (2010). Mann-Whitney *U* test. The Corsini encyclopedia of psychology. Hoboken, New Jersey, USA: John Wiley & Sons, Inc.

[ref36] Mellor K. C. Petrovska L. Thomson N. R. Harris K. Reid S. W. Mather A. E. (2019). Antimicrobial resistance diversity suggestive of distinct *Salmonella* Typhimurium sources or selective pressures in food-production animals. Front. Microbiol. 10:708. doi: 10.3389/fmicb.2019.00708, PMID: 31031720 PMC6473194

[ref37] Monte D. F. Sellera F. P. Lopes R. Keelara S. Landgraf M. Greene S. . (2020). Class 1 integron-borne cassettes harboring *bla*_CARB-2_ gene in multidrug-resistant and virulent *Salmonella* Typhimurium ST19 strains recovered from clinical human stool samples, United States. PLoS One 15:e0240978. doi: 10.1371/journal.pone.0240978, PMID: 33125394 PMC7598458

[ref38] Mulvey M. R. Boyd D. A. Olson A. B. Doublet B. Cloeckaert A. (2006). The genetics of *Salmonella* genomic island 1. Microbes Infect. 8, 1915–1922. doi: 10.1016/j.micinf.2005.12.028, PMID: 16713724

[ref39] Murray L. M. Hayes A. Snape J. Kasprzyk-Hordern B. Gaze W. H. Murray A. K. (2024). Co-selection for antibiotic resistance by environmental contaminants. NPJ Antimicrob Resist. 2:9. doi: 10.1038/s44259-024-00026-7, PMID: 39843965 PMC11721650

[ref40] NCBI (2016). National Library of Medicine (US), National Center for biotechnology information. Bethesda, MD: NCBI.

[ref41] Payne M. Kaur S. Wang Q. Hennessy D. Luo L. Octavia S. . (2020). Multilevel genome typing: genomics-guided scalable resolution typing of microbial pathogens. Euro Surveill. 25:1900519. doi: 10.2807/1560-7917.ES.2020.25.20.1900519 32458794 PMC7262494

[ref42] Pelé J. Bécu J.-M. Abdi H. Chabbert M. (2012). Bios2mds: an R package for comparing orthologous protein families by metric multidimensional scaling. BMC Bioinformatics. 13, 1–7. doi: 10.1186/1471-2105-13-133 22702410 PMC3403911

[ref43] Plumb I. Fields P. I. Bruce B. (2024). Salmonellosis, nontyphoidal. CDC yellow book 2024. Available online at: https://wwwnc.cdc.gov/travel/yellowbook/2024/infections-diseases/salmonellosis-nontyphoidal (accessed June 4, 2024).

[ref44] Rousseeuw P. J. (1987). Silhouettes: a graphical aid to the interpretation and validation of cluster analysis. J. Comput. Appl. Math. 20, 53–65. doi: 10.1016/0377-0427(87)90125-7

[ref45] Rozwandowicz M. Brouwer M. Fischer J. Wagenaar J. Gonzalez-Zorn B. Guerra B. . (2018). Plasmids carrying antimicrobial resistance genes in Enterobacteriaceae. J. Antimicrob. Chemother. 73, 1121–1137. doi: 10.1093/jac/dkx488, PMID: 29370371

[ref46] Ruegg P. L. (2022). Realities, challenges and benefits of antimicrobial stewardship in dairy practice in the United States. Microorganisms. 10:1626. doi: 10.3390/microorganisms10081626, PMID: 36014044 PMC9415423

[ref47] SciPy Documentation (2023). Available online at: https://docs.scipy.org/doc/scipy-1.10.1/index.html

[ref48] Segerman B. (2012). The genetic integrity of bacterial species: the core genome and the accessory genome, two different stories. Front. Cell. Infect. Microbiol. 2:116. doi: 10.3389/fcimb.2012.00116 22973561 PMC3434323

[ref49] Sherry N. L. Horan K. A. Ballard S. A. Gonҫalves da Silva A. Gorrie C. L. Schultz M. B. . (2023). An ISO-certified genomics workflow for identification and surveillance of antimicrobial resistance. Nat. Commun. 14:60. doi: 10.1038/s41467-022-35713-4 36599823 PMC9813266

[ref50] Shrestha J. Zahra F. Jr P C. (2023). Antimicrobial stewardship. Treasure Island, FL: StatPearls. 34283434

[ref51] Souvorov A. Agarwala R. Lipman D. J. (2018). SKESA: strategic k-mer extension for scrupulous assemblies. Genome Biol. 19:153. doi: 10.1186/s13059-018-1540-z, PMID: 30286803 PMC6172800

[ref52] Sun H. Wan Y. Du P. Bai L. (2020). The epidemiology of monophasic *Salmonella*. Typhimurium Foodborne Pathog. Dis. 17, 87–97. doi: 10.1089/fpd.2019.2676, PMID: 31532231

[ref53] Tyson G. H. Li C. Hsu C.-H. Ayers S. Borenstein S. Mukherjee S. . (2020). The *mcr*-*9* gene of *Salmonella* and *Escherichia coli* is not associated with colistin resistance in the United States. Antimicrob Agents Ch. 64, 520–573. doi: 10.1128/AAC.00573-20 PMC7526823 32513803

[ref54] Van Puyvelde S. Pickard D. Vandelannoote K. Heinz E. Barbé B. de Block T. . (2019). An African *Salmonella* Typhimurium ST313 sublineage with extensive drug-resistance and signatures of host adaptation. Nat. Commun. 10:4280. doi: 10.1038/s41467-019-11844-z, PMID: 31537784 PMC6753159

[ref55] VanderWeele T. J. Mathur M. B. (2019). Some desirable properties of the Bonferroni correction: is the Bonferroni correction really so bad? Am. J. Epidemiol. 188, 617–618. doi: 10.1093/aje/kwy250, PMID: 30452538 PMC6395159

[ref56] Williamson D. A. Lane C. R. Easton M. Valcanis M. Strachan J. Veitch M. G. . (2018). Increasing antimicrobial resistance in nontyphoidal *Salmonella* isolates in Australia from 1979 to 2015. Antimicrob Agents Ch. 62, 2012–2017. doi: 10.1128/AAC.02012-17 PMC5786757 29180525

[ref57] World Health Organization (2023). GLASS manual for antimicrobial resistance surveillance in common bacteria causing human infection. Geneva: World Health Organization.

