# Supplementary material for: Genomic dissection of the antimicrobial resistance epidemiology of Salmonella Typhimurium
Source: Front Microbiol. 2025 Nov 20;16:1683995. doi: 10.3389/fmicb.2025.1683995 (PMC12675436; doi:10.3389/fmicb.2025.1683995)
Supplement: Supplementary file 2 [file Supplementary_file_1.docx]

**Supplementary**

**Supplementary Results**

## Resistance prediction

Ciprofloxacin is often the first choice for treatment of severe *Salmonella* infections in humans (1), and hence the prediction of resistance to this antibiotic in STm is important. However, the prediction of ciprofloxacin resistance from genotype is complex (2). The complexity arises because the basis of resistance appears additive, with resistance increasing based on the number of genetic mechanisms present (2). Moreover, ciprofloxacin MICs cover a wide numeric range (2, 3), while clinical standards define discrete MIC breakpoints to define isolates as susceptible, intermediate and resistant, which can vary between standards. For example, in 2020 the Clinical & Laboratory Standards Institute (CLSI) defined three categories of ciprofloxacin resistance for *Salmonella*, namely susceptible (MIC <=0.06 µg/mL), intermediate (MIC 0.12 µg/mL-0.5µg/mL) and resistant (MIC ≥1 µg/mL) (4). In contrast, the European Committee on Antimicrobial Susceptibility Testing (EUCAST) only defines two categories, susceptible and resistant, with MIC breakpoints of <=0.06 mg/L and >0.06 mg/L, respectively (5).

abritAMR was developed and validated against CLSI (2020) (4) and predicts three categories of ciprofloxacin resistance, namely susceptible, intermediate and resistant (6). In abritAMR reporting logic, the absence of any resistance mechanism is coded as susceptible, the presence of one mechanism as intermediate, and the presence of two or more mechanisms as resistant (6). While this approach is grounded in the well-documented additive nature of ciprofloxacin resistance mechanisms (2), it may oversimplify the true complexity of resistance phenotypes where multiple resistance mechanisms do not always correlate linearly with clinical resistance outcomes. For example, in our dataset 60 isolates carrying two genes, *oqxA* and *oqxB*, were classified as ciprofloxacin resistant by abritAMR according to it’s reporting logic. However, these genes encode a single efflux pump (OqxAB), and prior studies have shown that the present of these two genes alone confers only low-level resistance to ciprofloxacin in STm (7, 8). Although further refinement of abritAMR’s predictions may be made to improve accuracy, we chose to retain its unmodified outputs to maintain consistency with other users of the tool. We acknowledge the limitations in predicting ciprofloxacin resistance using abritAMR and consider further refinements as a future task for the developers of the tool.

Throughout this paper, when performing any two-category analyses (e.g., calculating a resistance score), we grouped ciprofloxacin-intermediate isolates with resistant isolates, rather than susceptible isolates. The intermediate category (characterized by elevated MIC) has both clinical and epidemiological significance. Epidemiologically, monitoring of intermediate isolates remains critical for AMR surveillance, as increasing trends in this category may serve as early indicators of emerging resistance. Clinically, isolates in the intermediate category have been associated with lower treatment success rates compared to susceptible isolates (4), and systemic infections caused by isolates with any detectable resistance mechanism were associated with poor treatment response (5). Nevertheless clinical success may be likely if antibiotic exposure is optimized (e.g., higher doses, increased frequency, longer duration of treatment, or altered administration route) (5). Clinically, the intermediate category becomes particularly important when an isolate is resistant to all other available antibiotics, limiting treatment options. Therefore, we grouped intermediate and resistant isolates together for any two-category analysis but subsequently analysed them separately thus highlighting the distinct intermediate and resistant epidemiology.

In addition to prediction of resistance to the antibiotics listed above, abritAMR reports colistin resistance mechanisms and 16S rRNA methylase genes, which typically give high level resistance to aminoglycosides, but does not provide phenotypic interpretation. In our dataset, 219 isolates have genes or mutations potentially conferring resistance to colistin (**Table S1)** while only 44 isolates have an 16S rRNA methylase gene (**Table S2**). For colistin, we interpreted the presence of any mutations or an *mcr* gene, except *mcr-9* (**Table S1**), as resistant (9). 16S rRNA methylase genes generally confer high-level resistance to multiple aminoglycosides (including gentamicin, amikacin, and kanamycin) (10) but abritAMR did not validate this set of genes for phenotypic prediction. While we could validate the prediction of susceptibility (due to absence of genes), the dataset did not include any isolates with a 16S rRNA methylase gene which were also phenotypically resistant (by AST) for validation of prediction of resistance (**Table S3**). Therefore, predicted resistance conferred by these genes is not included in this paper.

## Resistance to any antibiotic

Yearly variation in predicted resistance was observed in each location (**Figure S2**). A comparison of the proportion of resistant isolates in the UK and USA between 2015-2018 and 2019-2022 revealed opposite trends. In the UK, the proportion of resistant isolates significantly decreased from 64% ($\bar{R_{s}}$=2.5) to 55% ($\bar{R_{s}}$=2.2), whereas in the USA, it significantly increased from 42% ($\bar{R_{s}}$=1.4) to 48% ($\bar{R_{s}}$=1.6). In addition to these shifts, statistical analysis confirmed that the mean resistance score ($\bar{R_{s}}$) also significantly decreased in the UK, and increased in the USA, over the two timeframes. However, when comparing the mean resistance score of isolates resistant to at least one antibiotic ($\bar{R_{s,\boldsymbol{R}}}$) no significant differences were observed between the two timeframes for either country. In the UK, $\bar{R_{s,\boldsymbol{R}}}$ remained stable at 4 in 2015-2018 and 4.1 in 2019-2022. Similarly, in the USA, $\bar{R_{s,\boldsymbol{R}}}$ was 3.4 in both timeframes. Lastly, a comparison of the UK and the USA isolates in 2019-2022 showed that the UK isolates had both a significantly higher $\bar{R_{s}}$ and a significantly higher $\bar{R_{s,\boldsymbol{R}}}$.

## Resistance within MGT1 (MLST) STs for individual antibiotics

Resistance to individual antibiotics was also compared between STs **(Figure S3**). As expected, ST34 isolates have higher resistance to most antibiotics (11 of 14) compared to ST19, including the currently prescribed antibiotics ciprofloxacin (1% higher levels of resistance, 2% higher levels of intermediate resistance), azithromycin (2% higher), and the last-line antibiotic colistin (2% higher). The other antibiotics that ST34 isolates also show higher resistance to are tetracycline (by 58%), streptomycin (by 59%), ampicillin (by 60%), sulfathiazole (by 58%), trimethoprim (by 8%), trimethoprim-sulfathiazole (by 7%), kanamycin (by 3.2%) and gentamicin (by 1.3%). In contrast, ST19 isolates had higher resistance to only two antibiotics, namely the currently prescribed cefotaxime (by 2%) and chloramphenicol (by 2%).

To understand any variability within an ST by location, resistance to each antibiotic was compared between isolates from the UK and the USA for 2019-2022, (**Figure S4**). ST19 isolates from the UK had significantly higher resistance to six antibiotics, including ampicillin (by 10%) and ciprofloxacin (intermediate resistance 7% higher). ST19 isolates from the USA also had higher resistance to five antibiotics, including cefotaxime (by 6%). ST34 isolates from the UK had higher resistance to eight antibiotics, and the USA ST34 isolates had higher resistance to four antibiotics, including ciprofloxacin (resistance 1% higher, intermediate resistance 12% higher) and cefotaxime (by 4%).

Distribution of the ASSuT resistance pattern

We determined the proportion of ST34 isolates which had the previously observed ASSuT phenotypic resistance pattern, attributed to the presence of *bla*_TEM-1_, *strAB*, *sul2*, and *tet*(B). In the complete dataset, 10% (6,307 isolates) carried this specific gene set. The majority of these isolates (91%, 5,729) are MGT1 ST34. Within ST34, 63% (5,729 of 9,122 isolates) have this genotypic resistance pattern (**Figure S5**). In addition to resistance conferred by the ASSuT gene set, the next most common resistance observed was to trimethoprim (and hence trimethoprim-sulfathiazole), found in 7.7% of ST34 isolates with the ASSuT genotype. Among the trimethoprim-resistant isolates, *dfrA12* was the most frequently detected gene (present in 60% of these isolates), followed by *dfrA1* (28%).

Distribution of the ACSSuT resistance pattern

We determined the proportion of DT104 (MGT3 ST11) isolates that had the previously observed ACSSuT phenotypic resistance pattern attributed to the presence of *bla*_CARB-2_, *floR, aadA2*, *sul1* and *tet*(G*)*. In the complete dataset, 1% (639 isolates) carry this specific gene set. The majority of these isolates (67%, 426 in total) are in MGT3 ST11, but only 8.8% (426 of 4,852) of MGT3 ST11 have this genotypic resistance pattern (**Figure S6**). In addition to resistance conferred by the ACSSuT gene set, intermediate resistance to ciprofloxacin was the next most common resistance observed, found in 14% of MGT3 ST11 isolates with the ACSSuT genotype. Among the ciprofloxacin-intermediate isolates, a single mutation in the *gyrA* gene, resulting in the D87N mutation in GyrA, was the most frequently detected mutation found in 61.7% of these isolates. DT104 isolates with this mutation have been previously observed (11). Additionally, variations in the ACSSuT gene set in DT104 isolates have also been previously observed; the ACSSuT gene set is present on the SG1, which is conducive to gene mobility and hence variability (12).

### Selection of the 80% cutoff to identify MGT STs defined as resistant (or intermediate/resistant to ciprofloxacin)

An 80% cutoff was selected to define MGT STs as resistant or ciprofloxacin-intermediate, meaning that at least 80% of isolates within an MGT ST must be predicted to be resistant to a given antibiotic (or intermediate to ciprofloxacin) for that ST to be classified as a resistant (or ciprofloxacin-intermediate) MGT ST. We tested five thresholds for this purpose (50%, 60%, 70%, 80% and 90%) and chose 80%, based on recall and precision trade-offs. At this threshold the recall, i.e. the number of resistant (resistant/intermediate for ciprofloxacin) isolates included in MGT STs defined as resistant (or resistant/intermediate for ciprofloxacin) was considerably higher compared to 90% for several antibiotics (particularly tetracycline, streptomycin, ampicillin and sulfathiazole). At lower thresholds further large differences in recall were not seen (**Figure S8a**). However, precision decreased at lower thresholds i.e. the number of susceptible isolates included in MGT STs defined as resistant (resistant/intermediate for ciprofloxacin) increased (**Figure S8b**).

### MGT STs for the ASSuT resistance pattern

Only a small proportion of isolates (8.8%) within MGT3 ST11 have the ACSSuT resistance pattern, but 63% of ST34 isolates have the ASSuT resistance pattern. Given this, we investigated whether MGT analysis could be useful for identifying MGT STs associated with the ASSuT resistance genes *bla*_TEM-1_, *strAB*, *sul2*, and *tet*(B) using an approach similar to that applied for defining resistant MGT STs. For ease of reference, we refer to such STs as “marked” MGT STs.

We assessed whether an optimal threshold could be identified for defining marked MGT STs but no clear threshold was found (**Figure S10**). At 50% and 60%, precision was too low, i.e., many isolates not carrying the gene set were incorrectly included in the marked MGT STs (**Figure S11**). At 70%, 80%, and 90%, while fewer isolates without the resistance gene set were included, a large proportion of isolates carrying the genes were excluded, leading to low recall (**Figure S11**). At the optimal threshold of 70%, 48.5% of isolates carrying the specific resistance gene pattern were classed into 80 marked MGT STs. This diversity in MGT STs suggests that the genes of the specified set are mobile. Previously, the gene set has been demonstrated to be on a chromosomal resistance island (13) comprising two distinct regions: region 1 (containing *bla*_TEM-1_, *strAB* and *sul2*) and region 2 (containing *tet*(B)). Both regions are flanked by IS*26* elements (13), which are known to mediate gene mobility (including both gene acquisition and deletions) (14), suggesting the source of variability in these isolates.

## MGT STs defined as resistant to important antibiotics

**Azithromycin**

Three MGT STs were defined as azithromycin resistant, and these included 78 of 629 (12%) of azithromycin resistant isolates and three susceptible isolates (**Figure S9**, **Supplementary Dataset 1**). Among these, only MGT5 ST4133 (15 isolates) had year metadata and were collected in 2017, mostly from the UK (**Supplementary Dataset 1**). Three originated from humans, with others missing source metadata. This ST belongs to MGT1 ST34, and the *mph*(A) gene explains the prediction of resistant for all isolates. The other two STs are MGT5 ST3370, which belongs to MGT1 ST313, where *mph*(A) also explains the prediction of resistant, and MGT5 ST5010, which belongs to MGT1 ST102, where the AcrB R717Q mutation explains the prediction of resistant.

The majority of isolates predicted to be azithromycin resistant (88%, 551 of 629) were not classified in MGT STs defined as azithromycin resistant. These isolates were found in various regions, including the UK (181 isolates), USA (95 isolates) and Other (168 isolates), with the rest missing country metadata. These resistant isolates were distributed across 12 MGT1 STs, 44 MGT2 STs and 131 MGT3 STs. These isolates were from 1988-2022, with 45 isolates collected in 2021-2022. Among these 551 isolates, 28 distinct resistance determinants, including genes, incomplete genes (≤10% sequence missing), mutations, or combinations, were identified. The *mph*(A) gene was the most prevalent resistance mechanism, found in 40% of isolates. In 2021–2022, isolates carrying *mph*(A) were detected across multiple countries, whereas isolates carrying *mef*(B) were exclusively found in the UK. The prediction of azithromycin resistance across diverse MGT STs, rather than specific clonal lineages, suggests that it is likely driven by horizontal gene transfer (*mph*(A) has often been detected on plasmids (15, 16)), along with selection pressures and independent mutation events.

**Colistin**

One MGT ST, MGT5 ST5868, was defined as resistant to colistin. However, only 14 of 219 (6.4%) isolates predicted to be colistin resistant belong to MGT5 ST5868 (**Figure S9**, **Supplementary Dataset 1**). All but one isolate in this MGT ST carry the *mcr-1.1* gene. All isolates in this ST were from 2020 and were predominantly from China.

The majority of isolates predicted as colistin resistant were not assigned to MGT STs defined as colistin resistant. These isolates were found across multiple regions, including the UK (76 isolates), USA (5 isolates), and Other (114 isolates), with the remainder (10 isolates) lacking country metadata. These resistant isolates were distributed across six MGT1 STs (79% in ST34), 12 MGT2 STs and 48 MGT3 STs. They were collected from 1984 to 2022, with 10 isolates collected in 2021-2022. A total of 18 distinct resistance determinants, including genes, incomplete genes (≤10% sequence missing) and mutations, were identified, with *mcr-1.1* being the most prevalent (55% of isolates). The fact that 93.6% of colistin-resistant isolates were not confined to a single resistant MGT ST suggests that colistin resistance is emerging through multiple independent genetic events (*mcr-1.1* has often been detected on plasmids in multiple species globally (17)) rather than being driven by clonal expansion alone.

Lastly, we present the epidemiology of the *mcr-9* gene shown not to confer resistance to colistin in *Salmonella* (9, 18), but it may confer reduced susceptibility to colistin when induced under experimental conditions (19). In total, 206 isolates were identified as carrying *mcr-9*, with 198 isolates containing the full-length gene. These isolates were detected over a broad timeframe, spanning 1995 to 2022, and were distributed across multiple regions, 60 isolates in the UK, 44 isolates in the USA, 86 isolates in Other, 16 isolates with missing country metadata. These *mcr-9* positive isolates were distributed across six MGT1 STs (65% in ST19), eight MGT2 STs, and 29 MGT3 STs.

**Meropenem**

Only 14 resistant isolates were predicted to be resistant to meropenem and no MGT STs were defined as meropenem resistant (**Figure S9**). Seven of the 14 isolates were collected in 2021-2022 and all have *bla*_OXA-48_, also found in five of seven earlier isolates, with the remaining two having *bla*_OXA-181_ or *bla*_NDM-5_. Five meropenem-resistant isolates from 2021-2022 originated in the UK and two in South Africa, while the remaining isolates lacked country information. These resistant isolates were distributed among three MGT1 sequence types: MGT1 ST19 (7 isolates), MGT1 ST34 (4 isolates), and MGT1 ST0 (3 isolates).

Reliability of resistance prediction

Most isolates from recent years that are predicted to be cefotaxime resistant have *bla*_CMY-2_, known to be carried by various plasmids in *Salmonella* (20). We assessed the reliability of this prediction by investigating whether the genome assembly approach or the pipeline used for prediction of resistance influenced the detection of *bla*_CMY-2_-like alleles. We analysed 437 isolates from the eight major MGT STs defined as cefotaxime resistant from 2021-2022. Twenty of these were predicted to be susceptible to cefotaxime by our pipeline. We compared abritAMR-based predictions using genome assemblies generated by two different tools: SKESA (21) and SPAdes (22). Notably, SKESA is also integrated into the MGT pipeline (23). All isolates identified as carrying *bla*_CMY-2_ using SKESA assemblies were also identified as having *bla*_CMY-2_ using SPAdes assemblies. However, using SPAdes assemblies a full-length *bla*_CMY-2_ gene was detected in additional five isolates and a partial *bla*_CMY-2_ (missing ≤10% of the sequence) in another five isolates. These findings suggest that the choice of genome assembly algorithm can influence the detection of resistance genes, with SPAdes potentially offering higher sensitivity for identifying both complete and partial gene sequences.

We then applied KMA (24), a *k*-mer based approach for matching and aligning raw-reads to a query sequence, to evaluate whether an assembly-free method improves gene detection. All isolates identified as carrying the full-length *bla*_CMY-2_ by SPAdes were also identified using KMA. However, three genomes where a partial gene was identified by SPAdes were found to carry the complete gene using KMA. Furthermore, using KMA, four additional isolates were identified as carrying *bla*_CMY-2_ (or a *bla*_CMY-2_-like allelle), one with an exact match to the reference, two with partial gene matches (missing ≤10% of the sequence), and one with mutations (100% coverage, 99% identity). Thus, of the 20 isolates predicted as susceptible using our pipeline, six isolates were consistently identified as lacking *bla*_CMY-2_ using all three methods, 10 had the complete gene, and four were either partial (≤10% missing sequence) or contained mutations, when using alternative approaches. Thus, reliance on SKESA assembly for resistance prediction using abritAMR may result in underreporting of resistance due to missed *bla*_CMY-2_ carriage.

The abritAMR tool relies on the identification of resistance mechanisms via AMRFinderPlus (25) and assigns a resistance interpretation based on the identified mechanisms. Regardless of whether SKESA or SPAdes assemblies were used, AMRFinderPlus detected *bla*_CMY-2_ in all 14 genomes noted above with the prediction discrepancy. However, when between 50-90% of the gene sequence was present, abritAMR classified the isolate as ‘susceptible’ leading to a discrepancy in prediction. Despite this, a potential underreporting of at most 14 genomes represents an error rate of at most 3%, which remains comparable to the overall accuracy of the resistance prediction, and hence acceptable in this analysis.

Most isolates from recent years that are predicted to be intermediately resistant to ciprofloxacin have mutations in *gyrA*, which encodes the essential protein DNA gyrase subunit A. This gene is part of the core genome of *Salmonella* (MGT8), and is also included in the MGT7 scheme (23). The presence of a core gene is highly conserved, and these genes can be reliably identified and genotyped in at least 95% of the isolates of the species (23). As *gyrA* is a core gene, any mutations in it can be reliably identified, including mutations that may confer resistance. Hence, the identification of MGT STs defined as intermediate based on the presence of resistance-conferring mutations in *gyrA* can be considered reliable. Furthermore, since *gyrA* is a core gene and forms part of the MGT7 and MGT8 schemes, any novel mutations relative to the reference genome result in the assignment of new STs at both these levels. However, in this analysis, lower-resolution MGT levels (MGT2 and MGT5) could effectively distinguish and identify ciprofloxacin-intermediate isolates. As per the MGT design (23), correlation at broader-resolution levels (compared to the MGT7 and MGT8 levels here) generally reflects that the mutation conferring intermediate resistance has been maintained over an extended timeframe in the associated genotypes.

## Supplementary methods

## Reliability of cefotaxime resistance prediction based on presence of *bla*_CMY-2_

To test the reliability of *bla*_CMY-2_ annotations, the sequences from isolates belonging to eight MGT STs were reassembled using SPAdes (22) (version 3.15.5) with the option ‘--isolate'. Then, abritAMR was run on the assemblies, followed by the ‘report’ option to generate an interpretation of resistance.

KMA (24) was used to match raw-reads against the *bla*_CMY-2_ sequence. We downloaded the *bla*_CMY-2_ sequence (RefSeq ID: NG_048814.1) from the NCBI Reference Gene Catalog (25). We indexed this sequence using KMA (24) (version 1.4.10) using the default settings. We then used KMA with default settings to match the reads to the indexed sequence.

## Supplementary figures

##

**Figure S1**. Temporal distribution of isolates in dataset. **a**. Complete dataset. **b-d**. Isolates from the UK, the USA and Other locations, 2015-2022.

**Figure S2**. Bootstrapped mean resistance scores and standard deviation in each year 2015-2022 for isolates from the UK, the USA and Other. The top row shows these statistics for all data in the three locations. The bottom row shows these statistics for isolates resistant to at least one antibiotic or intermediate to ciprofloxacin (i.e. excludes isolates susceptible to all antibiotics).

**Figure S3**. Percentages of isolates predicted to be resistant to 14 antibiotics, or intermediate to ciprofloxacin, in the four most frequent MGT1 STs and other STs. Bars outlined in blue are those for which that ST has the highest percentage of resistant (or ciprofloxacin-intermediate) isolates among all MGT1 STs. If ST19 or ST34 did not have the highest percentage of resistant (or ciprofloxacin-intermediate) isolates for that antibiotic, then a yellow box indicates which has the highest in this pair of STs. The resistance distributions were analyzed using the Binomial test, using a Bonferroni corrected *p*-value <0.0001.

**Figure S4**. Predicted resistance to 14 antibiotics in isolates from the UK and the USA across two time periods (2015-2018, 2019-2022) for MGT1 STs ST19, ST34 and other MGT1 STs. Antibiotics are listed from left to right in descending order of resistance in the complete dataset (see **Figure 1**), with the exception of colistin, which is shown at the end, as interpretation for prediction of resistance was not validated for this antibiotic. Red arrows indicate significantly higher resistance (and intermediate resistance for ciprofloxacin) in 2019-2022 compared to 2015-2018, green arrows indicate significantly lower resistance (and intermediate resistance for ciprofloxacin) in 2019-2022 compared to 2015-2018. A blue outline highlights antibiotics for which resistance (and intermediate resistance for ciprofloxacin) was significantly higher in the UK vs. the USA in 2019-2022. The proportions of resistant or ciprofloxacin-intermediate isolates were compared using the Binomial test. Significance was assessed at the Bonferroni corrected *p*-value < 0.0002.

**Figure S5**. Temporal distribution of ST34 isolates with the characteristic ASSuT gene set [*bla*_TEM-1_, *strAB*, *sul2*, *tet*(B)] (blue) vs. isolates that do not have this full set of genes set (grey). Isolates in either group may carry additional resistance genes or mutations.

**Figure S6**. Temporal distribution of MGT3 ST11 (DT104) isolates with the characteristic ACSSuT gene set [*bla*_CARB-2_, *floR*, *aadA2*, *sul1*, *tet*(G)] (blue) vs. isolates that do not have this full set of genes (grey). Isolates in either group may carry additional resistance genes or mutations.

**Figure S7**. Identification of MGT STs defined as resistant or ciprofloxacin-intermediate. For each antibiotic, the identification begins at MGT1, the broadest level of resolution. Any MGT1 STs containing more than a certain percentage (e.g. 80%) of isolates predicted to be resistant (or ciprofloxacin-intermediate) are designated as a resistant MGT ST or an intermediate MGT ST for ciprofloxacin. Isolates not assigned to such an MGT ST at this level are considered at the next MGT level. This process is repeated through all MGT levels until either all resistant isolates (or intermediate isolates for ciprofloxacin) are assigned to a corresponding MGT ST, or no further MGT levels remain. Since each successive MGT level provides finer resolution, isolates are divided into more specific STs at the higher levels.

**Figure S8**. Impact of different inclusion thresholds on the accuracy of classifying MGT STs defined as resistant or ciprofloxacin-intermediate. Thresholds of ≥50%, ≥60%, ≥70%, ≥80%, or ≥90% of resistant or ciprofloxacin-intermediate isolates in an MGT ST were tested for defining a “resistant MGT ST” for that antibiotic, or a “resistant or intermediate MGT ST” for ciprofloxacin. a. Proportion of resistant or ciprofloxacin-intermediate isolates included in MGT STs defined as resistant or ciprofloxacin-intermediate at each threshold. b. Proportion of susceptible isolates included in MGT STs defined as resistant or ciprofloxacin-intermediate at each threshold.

**Figure S9**. Inclusion of isolates in MGT STs defined as resistant or ciprofloxacin-intermediate. MGT STs defined as resistant or ciprofloxacin-intermediate are those with at least 80% of isolates predicted to be resistant to a given antibiotic, or intermediate to ciprofloxacin. Blue indicates resistant or ciprofloxacin-intermediate isolates in MGT STs defined as resistant or ciprofloxacin-intermediate, orange indicates resistant or ciprofloxacin-intermediate isolates not in MGT STs defined as resistant or ciprofloxacin-intermediate, and green indicates susceptible isolates in MGT STs defined as resistant or ciprofloxacin-intermediate. a. Counts. b. Percentages, scaled to the total count of resistant isolates for the given antibiotic, and includes intermediate isolates for ciprofloxacin.

**Figure S10**. Precision, recall, and F1-score values for the classification of ST34 isolates carrying the ASSuT gene set [*bla*_TEM-1_, *strAB*, *sul2*, *tet*(B)], versus those without it, into marked MGT STs at higher MGT levels within ST34. Marked MGT STs are MGT STs comprising mutually exclusive sets of isolates in which more than the specified percentage of isolates carry the ASSuT gene set. While at the thresholds of 50% and 60% the precision is low, at 70%, 80% and 90% the recall is low.

**Figure S11**. Evaluating the accuracy of MGT ST classification in grouping isolates based on the presence of the ASSuT gene set [*bla*_TEM-1_, *strAB*, *sul2*, *tet*(B)] at different thresholds for inclusion into marked MGT STs. Marked MGT STs are MGT STs comprising mutually exclusive sets of isolates in which more than the specified percentage of isolates carry the ASSuT gene set. At a given threshold (e.g., 50%) at least that percentage of isolates carrying the gene combination are included in an MGT ST. TP (True Positives), isolates correctly classified as carrying the resistance gene set in marked MGT STs; FP (False Positives), isolates that do not carry the resistance gene set but are included in marked MGT STs; FN (False Negatives), isolates that carry the resistance gene set but are excluded from marked MGT STs.

**Figure S12**. Temporal trends for major MGT STs defined as cefotaxime resistant in 2015-2022. Eight dominant trends are shown. Clusters were generated using the unsupervised *c*-means clustering algorithm, with the optimal number of clusters determined through a combination of the silhouette index and manual inspection of temporal delineation. Each cluster represents a distinct temporal pattern, with the bold black line indicating the weighted mean trend of all MGT STs within the cluster. Coloured lines represent the temporal profiles for individual MGT STs as the standardized count of isolates per year in that MGT ST. Clusters are arranged based on the timing of their first peak. The cluster header lists the number of MGT STs in the cluster, the total number of isolates within the cluster, the geographical distribution of these isolates, and the number of MGT STs defined as resistant to each antibiotic, and defined as resistant or intermediate to ciprofloxacin, listed in the following order: tetracycline, streptomycin, ampicillin, sulfathiazole, chloramphenicol, trimethoprim, trimethoprim-sulfathiazole, cefotaxime, kanamycin, gentamicin, azithromycin, ciprofloxacin, meropenem, colistin.

**Figure S13**. Temporal trends for major MGT STs defined as ciprofloxacin resistant or intermediate in 2015-2022. Seven dominant trends are shown. Clusters were generated using the unsupervised *c*-means clustering algorithm, with the optimal number of clusters determined through a combination of the silhouette index and manual inspection of temporal delineation. Each cluster represents a distinct pattern, with the bold black line indicating the weighted average of all MGT STs within the cluster. Coloured lines represent temporal profiles for individual MGT STs as the standardized count of isolates per year in that MGT ST. Clusters are arranged based on the timing of their first peak. The cluster header lists the number of MGT STs in the cluster, the total number of isolates in the cluster, the geographical distribution of these isolates, and the number of MGT STs defined as resistant to each antibiotic, or defined as resistant or intermediate to ciprofloxacin, listed in the following order: tetracycline, streptomycin, ampicillin, sulfathiazole, chloramphenicol, trimethoprim, trimethoprim-sulfathiazole, cefotaxime, kanamycin, gentamicin, azithromycin, ciprofloxacin, meropenem, colistin.

**Figure S14**. Characteristics of sixty MGT STs defined as cefotaxime resistant. **a**. Similarity of MGT STs based on cefotaxime resistance genes. MGT STs are divided into eight clusters according to shared resistance genes. **b**. Country distribution. Geographic distribution of isolates in each MGT ST. **c**. MGT ST size. The total number of isolates in each MGT ST.

**Figure S15**. Characteristics of fifty-three MGT STs defined as ciprofloxacin resistant or intermediate. **a**. Similarity of MGT STs based on common resistance genes or mutations. MGT STs are divided into 14 clusters according to shared resistance genes or mutations. **b**. Country distribution. Geographic distribution of isolates within each MGT ST. **c**. MGT ST size. The total number of isolates in each MGT ST. **d**. Predicted phenotypic resistance, intermediate or susceptibility to ciprofloxacin. The percentage of isolates predicted to be phenotypically resistant, intermediate or susceptible to ciprofloxacin based on their resistance mechanisms.

**Supplementary references**

1. I. Plumb PPF, and C. Beau Bruce. Salmonellosis, nontyphoidal. CDC Yellow Book 2024. 2024 [Available from: <https://wwwnc.cdc.gov/travel/yellowbook/2024/infections-diseases/salmonellosis-nontyphoidal>.

2. Conley ZC, Bodine TJ, Chou A, Zechiedrich L. Wicked: The untold story of ciprofloxacin. PLoS pathog. 2018;14(3):e1006805.

3. Chang M-X, Zhang J-F, Sun Y-H, Li R-S, Lin X-L, Yang L, et al. Contribution of different mechanisms to ciprofloxacin resistance in *Salmonella* spp. Front Microbiol. 2021;12:663731.

4. CLSI M100. Performance Standards for Antimicrobial Susceptibility Testing. 30 ed2020.

5. “The European Committee on Antimicrobial Susceptibility Testing.”. Breakpoint tables for interpretation of MICs and zone diameters. 2025 [Version 15.0:[Available from: <https://www.eucast.org/fileadmin/src/media/PDFs/EUCAST_files/Breakpoint_tables/v_15.0_Breakpoint_Tables.pdf>.

6. Sherry NL, Horan KA, Ballard SA, Gonҫalves da Silva A, Gorrie CL, Schultz MB, et al. An ISO-certified genomics workflow for identification and surveillance of antimicrobial resistance. Nat Commun. 2023;14(1):60.

7. Chan BK-w, Wong MH-y, Chan EW-c, Chen S. Transcriptional regulation and functional characterization of the plasmid-borne *oqxAB* genes in *Salmonella* Typhimurium. Microbiol Spectr. 2022;10(2):e02170-21.

8. Li J, Zhang H, Ning J, Sajid A, Cheng G, Yuan Z, et al. The nature and epidemiology of OqxAB, a multidrug efflux pump. Antimicrob Resist Infect Control. 2019;8:1-13.

9. Tyson GH, Li C, Hsu C-H, Ayers S, Borenstein S, Mukherjee S, et al. The *mcr*-*9* gene of *Salmonella* and *Escherichia coli* is not associated with colistin resistance in the United States. Antimicrob Agents Chemother. 2020;64(8):10.1128/aac. 00573-20.

10. Krause KM, Serio AW, Kane TR, Connolly LE. Aminoglycosides: an overview. Cold Spring Harb Perspect Med. 2016;6(6):a027029.

11. Mølbak K, Baggesen DL, Aarestrup FM, Ebbesen JM, Engberg J, Frydendahl K, et al. An outbreak of multidrug-resistant, quinolone-resistant *Salmonella enterica* serotype Typhimurium DT104. N Engl J Med. 1999;341(19):1420-5.

12. Mulvey MR, Boyd DA, Olson AB, Doublet B, Cloeckaert A. The genetics of *Salmonella* genomic island 1. Microbes Infect. 2006;8(7):1915-22.

13. Lucarelli C, Dionisi AM, Filetici E, Owczarek S, Luzzi I, Villa L. Nucleotide sequence of the chromosomal region conferring multidrug resistance (R-type ASSuT) in *Salmonella* Typhimurium and monophasic *Salmonella* Typhimurium strains. J Antimicrob Chemother. 2012;67(1):111-4.

14. Harmer CJ, Hall RM. IS*26* and the IS*26* family: versatile resistance gene movers and genome reorganizers. Microbiol Mol Biol Rev. 2024;88(2):e00119-22.

15. Wang H, Cheng H, Huang B, Hu X, Chen Y, Zheng L, et al. Characterization of resistance genes and plasmids from sick children caused by *Salmonella enterica* resistance to azithromycin in Shenzhen, China. Front Cell Infect Microbiol. 2023;13:1116172.

16. Stein M, Brinks E, Loop J, Habermann D, Cho G-S, Franz CM. Antibiotic resistance plasmids in *Enterobacteriaceae* isolated from fresh produce in northern Germany. Microbiol Spectr. 2024;12(11):e00361-24.

17. Wang R, Van Dorp L, Shaw LP, Bradley P, Wang Q, Wang X, et al. The global distribution and spread of the mobilized colistin resistance gene *mcr-1*. Nat Commun. 2018;9(1):1179.

18. Carroll LM, Gaballa A, Guldimann C, Sullivan G, Henderson LO, Wiedmann M. Identification of novel mobilized colistin resistance gene *mcr*-*9* in a multidrug-resistant, colistin-susceptible *Salmonella enterica* serotype Typhimurium isolate. mBio. 2019;10(3):10.1128/mbio. 00853-19.

19. Kieffer N, Royer G, Decousser J-W, Bourrel A-S, Palmieri M, Ortiz De La Rosa J-M, et al. *mcr-9*, an inducible gene encoding an acquired phosphoethanolamine transferase in *Escherichia coli*, and its origin. Antimicrob Agents Chemother. 2019;63(9):10.1128/aac. 00965-19.

20. Bortolaia V, Hansen KH, Nielsen CA, Fritsche TR, Guardabassi L. High diversity of plasmids harbouring *bla*_CMY-2_ among clinical *Escherichia coli* isolates from humans and companion animals in the upper Midwestern USA. J Antimicrob Chemother. 2014;69(6):1492-6.

21. Souvorov A, Agarwala R, Lipman DJ. SKESA: strategic k-mer extension for scrupulous assemblies. Genome Biol 2018;19(1):153.

22. Bankevich A, Nurk S, Antipov D, Gurevich AA, Dvorkin M, Kulikov AS, et al. SPAdes: a new genome assembly algorithm and its applications to single-cell sequencing. J Comput Biol. 2012;19(5):455-77.

23. Payne M, Kaur S, Wang Q, Hennessy D, Luo L, Octavia S, et al. Multilevel genome typing: genomics-guided scalable resolution typing of microbial pathogens. Euro Surveill. 2020;25(20):1900519.

24. Clausen PT, Aarestrup FM, Lund O. Rapid and precise alignment of raw reads against redundant databases with KMA. BMC Bioinformatics. 2018;19:1-8.

25. Feldgarden M, Brover V, Gonzalez-Escalona N, Frye JG, Haendiges J, Haft DH, et al. AMRFinderPlus and the Reference Gene Catalog facilitate examination of the genomic links among antimicrobial resistance, stress response, and virulence. Sci Rep. 2021;11(1):12728.
